# Sialic acid linkage differentiation of glycopeptides using capillary electrophoresis – electrospray ionization – mass spectrometry

**DOI:** 10.1038/s41598-017-03838-y

**Published:** 2017-06-16

**Authors:** Guinevere S. M. Kammeijer, Bas C. Jansen, Isabelle Kohler, Anthonius A. M. Heemskerk, Oleg A. Mayboroda, Paul J. Hensbergen, Julie Schappler, Manfred Wuhrer

**Affiliations:** 1 Leiden University Medical Center, Center for Proteomics and Metabolomics, Leiden, The Netherlands; 2 University of Applied Sciences Leiden, Leiden, The Netherlands; 3 University of Geneva, University of Lausanne, School of Pharmaceutical Sciences, Geneva, Switzerland

## Abstract

Sialylation is a glycosylation feature that occurs in different linkages at the non-reducing end of a glycan moiety, the linkage isomers are often differentially associated with various biological processes. Due to very similar physico-chemical properties, the separation of isomeric sialylated glycopeptides remains challenging but of utmost importance in the biomedicine and biotechnology, including biomarker discovery, glyco-engineering and biopharmaceutical characterization. This study presents the implementation of a high-resolution separation platform based on capillary electrophoresis – mass spectrometry (CE–MS) allowing for the selective analysis of α2,3- and α2,6-sialylated glycopeptides. These differentially linked glycopeptides showed an identical fragmentation pattern (collision induced dissociation) but different electrophoretic mobilities, allowing for baseline separation of the different linkages without the need for an extensive sample preparation. The different migration behavior between the two moieties was found to correlate with differences in pK_a_ values. Using a novel methodology adapted from the so-called internal standard CE approach, a relative difference of 3.4·10^−2^ in pK_a_ unit was determined. This approach was applied for the analysis of tryptic glycopeptides of prostate specific antigen, which shows highly complex and heterogeneous glycosylation. The developed platform therefore appears attractive for the identification of differentially linked sialic acids that may be related to pathological conditions.

## Introduction

Glycosylation is one of the most complex post-translational modifications of proteins that affects protein folding, stability, half-life, protein-protein interactions, signaling and trafficking^1–3^. Carbohydrate moieties are attached to the peptide backbone either via the nitrogen atom of the side-chain of asparagine (*N*-linked) with the sequence *N-X-S/T* (*X ≠ P*) or via an oxygen atom (*O*-linked) of serine or threonine. Next to the diversity in site occupancy (macroheterogeneity), a large variety in glycan structures may also be observed at a given glycosylation site (microheterogeneity).

Changes in glycosylation may be associated with various factors, including age and pathophysiological conditions^4–7^. In certain disease settings, specific glycan features such as fucosylation, bisection, galactosylation or sialylation are known to be affected^8, 9^. Sialylation, *i*.*e*., a process by which sialic acids are attached to glycans, occurs on different monosaccharides and in different linkage positions, namely α2,3, α2,6 or α2,8. The motifs are located at the non-reducing end of the glycans, creating binding sites for multiple human lectins, toxins and pathogens^10^. Sialic acids are involved in numerous pathophysiological processes. For instance, α2,3-linked sialic acids have shown to be associated with malignancy in several types of cancers via formation of sialyl-Lewis^x^ structures (attachment of an α1–3 linked-fucose to an antennary *N*-acetylglucosamine) which are linked to metastasis progression^11–13^. Furthermore, α2,6-linked sialic acids are presumed to be involved in blocking galectin binding and enhancing the tumor cell survival^14–16^.

Glycosylation, and especially changes thereof, has become of great importance due to the potential use of glycopeptides as clinical biomarkers^17–19^. Similarly, the structural characterization of protein-linked glycans is essential in the biopharmaceutical industry to ensure the safety and efficacy of novel therapeutics^1, 2, 20, 21^. In recent years, molecular engineering of carbohydrates, referred to as glyco-engineering, has emerged in this field to enhance efficacy and design functional properties of monoclonal antibodies (mAbs)^22–24^. Notably, terminal carbohydrate moieties on glycan chains are often affected by subtle changes in the cell culture environment^20, 21, 25^.

The characterization of protein glycosylation often requires information about site occupancy as well as the glycan structures. This information is often obtained via proteolytic cleavage and subsequent glycopeptide analysis by mass spectrometry (MS)^26, 27^. However, an exhaustive characterization of sialic acid linkages of glycopeptides using MS remains rather challenging. Only recently, ion mobility mass spectrometry has demonstrated to allow for the separation of glycopeptide sialic acid linkage isomers^28^. Likewise, the differentiation between the differently linked sialic acids of immunoglobulin G (IgG) Fragment crystallizable (Fc) glycopeptides has been achieved using a linkage-specific derivatization method prior to MS analysis^29^. Nonetheless, such methods are not broadly accessible and there is a clear need for the differentiation of sialic acid isomers in conjunction with MS detection.

In this study, we present a novel approach based on capillary electrophoresis (CE) – electrospray ionization (ESI)-MS for glycopeptides analysis, enabling baseline separation of the α2,3- and α2,6-sialylated isomers without the need for any sample pre-treatment. The CE–ESI-MS method was developed using IgG Fc glycopeptides derived from recombinant IgG which contains only α2,3-sialylated glycopeptides^20, 30^, as well as IgG Fc glycopeptides derived from human plasma IgG, which only contains α2,6-sialylated glycopeptides^31^. The observed difference in electrophoretic mobilities was further investigated by determining the difference in pK_a_ values of two representative compounds, namely, α2,3-sialyllactose and α2,6-sialyllactose. The relative difference in acidity constant was assessed using a novel methodology based on the internal standard CE approach first proposed by Fuguet *et al*.^32^, identifying differences in acidity between sialic acid isomers as one of the plausible causes for sialic acid isomer separation. Finally, the developed platform was applied to discriminate sialic acid linkage isomers of tryptic glycopeptides of prostate specific antigen (PSA)^33^.

## Results and Discussion

### Analysis of IgG Fc glycopeptides using CE–ESI-MS

**Figure 1:**
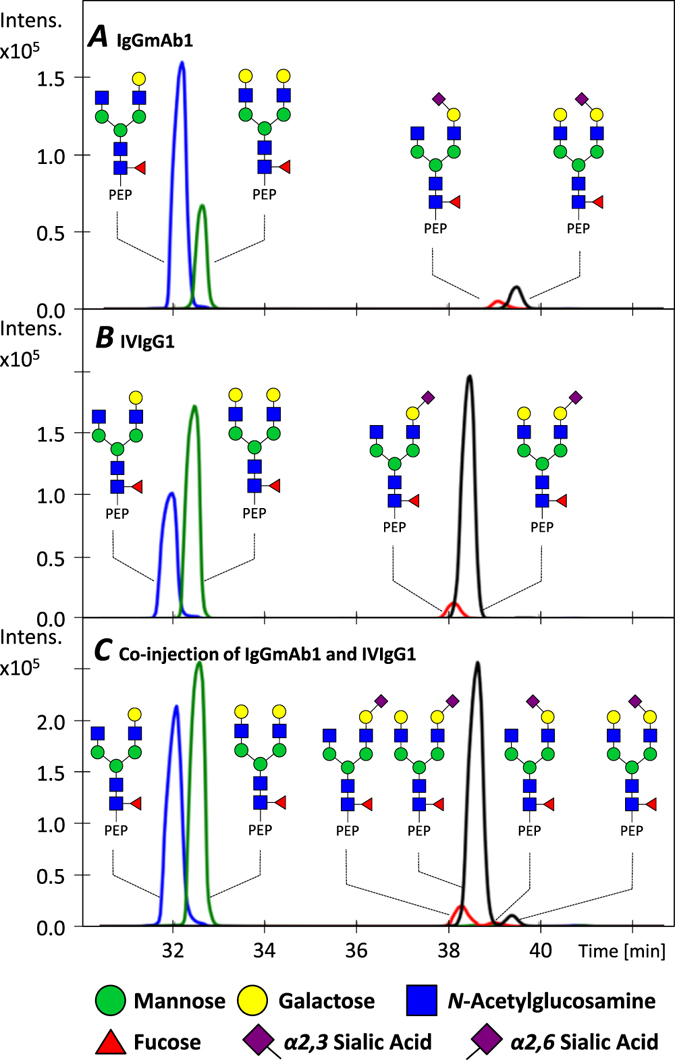
Extracted ion electropherograms (EIEs) of IgGmAb1 and IVIgG1 glycopeptides obtained with CE–ESI-MS after targeted alignment. (**A**) EIEs of IgGmAb1 glycopeptides derived from CHO cells, (**B**) EIEs of IVIgG1 retrieved from human plasma and (**C**) EIEs of a co-injection of IgGmAb1 and IVIgG1. The “PEP” label illustrates the tryptic peptide sequence EEQY**N**STYR to which the glycan is attached.

A sheathless CE–ESI-MS platform was used for the analysis of Fc glycopeptides derived from pooled intravenous IgG (IVIgG) and IgG1 monoclonal antibody (IgGmAb) produced in Chinese Hamster Ovary (CHO) cells. In order to correct for the significant differences observed in migration times (t_m_), a targeted alignment of the data was applied using an internal standard. Figure 1 illustrates the analysis of IgGmAb1 (Fig. 1A) and IVIgG (Fig. 1B) after alignment. For both samples, the extracted ion electropherograms of the non-sialylated glycopeptides of subclass 1 (G1F and G2F) showed the same apparent mobility while different t_m_ were obtained for the mono-sialylated species (G1FS and G2FS). IgG Fc glycopeptides derived from CHO cells are well-known to contain only α2,3-linked sialic acids while IgG Fc glycopeptides derived from human plasma are expected to only contain α2,6-linkages^20, 30, 31^. The co-injection of IgGmAb1 and IVIgG1 glycopeptides (Fig. 1C) emphasized this difference in apparent mobilities for α2,3- versus α2,6-linked sialylated species, where α2,3-linked mono-sialylated and α2,6-linked mono-sialylated glycopeptides were baseline resolved. The differentiation between both linkages is highly relevant since α2,3 and α2,6 linkages may have significantly different biological effects^29^. CE is not the only separation platform that enables separation of sialylated isomers: zwitterionic -HILIC and porous graphitized carbon (PGC)-LC have proven to be suitable for separation of glycan species with isomeric sialic acid linkages^34, 35^. Specific fragmentation features have been reported for anionic glycans, enabling the distinction between isomers^36, 37^. However, many other conventional positive ionization mode MS(/MS) approaches do not provide sufficient differentiation between α2,3- and α2,6-linked sialylated species due to an identical exact mass as well as similar fragmentation patterns using collision induced dissociation^38^.

Our preliminary data obtained for the analysis of IgG Fc glycopeptides using CE–ESI-MS showed different electrophoretic mobilities of sialic acid linkage isomers, implying a different charge-to-size ratio that may be used to discriminate both α2,3- and α2,6-linked sialylated species. This difference may be either caused by differences in the hydrodynamic radius of the analytes and/or by a difference in acidic constant between α2,3- and α2,6-linked sialic acids. To the best of our knowledge, only a general pK_a_ value for both linkages (pK_a_ *ca*. 2.6–2.9) has been described so far^39–41^. Therefore, the potential difference in pK_a_ values between the two differently linked sialic acids was further investigated using two representative compounds, namely, α2,3-sialyllactose and α2,6-sialyllactose.

### pK_a_ Differences between α2,3- and α2,6- Sialylated Lactose

The *in silico* prediction as well as the experimental determination of the pK_a_ values are described in Supplementary Information, Section S-1 ^42^. As summarized in Table 1, both approaches were not sufficient to demonstrate a significant difference in the pK_a_ values of α2,3- and α2,6-linked sialyllactose due to the relative close values and the associated error with the measurements. In order to further explore the expected difference in pK_a_ values and to enable a clear differentiation between both pK_a_ with minimized measurement errors, a novel methodology based on the so-called internal standard (IS)-CE approach was implemented for the determination of the relative difference in pK_a_ values. This IS-CE approach, first proposed by Fuguet *et al*.^32^, is based on the assessment of acidity constants using CE with concomitant injection of the analyte of interest and an IS. The pK_a_ values of the latter is known and its selection is based on its close similarity in nature and pK_a_ value to the analyte of interest. Here, this procedure was adapted to assess the relative difference in pK_a_ unit where one of the sialyllactose acted as the IS and the other one as the analyte of interest. The relative difference was estimated by calculating the mean limiting mobilities (migration under fully deprotonated form, namely, at pH 6.0, *N* = 2) and mean effective mobilities (migration under partially ionized form, namely, at pH 2.0, *N* = 2) for both analytes (α2,3- and α2,6-linked sialyllactose) injected simultaneously therefore no alignment was needed (S-7 Supplementary Information, Fig. S-1). Mean limiting mobilities for both compounds were found to be −6.0·10^−5^ cm^2^ V^−1^ s^−1^ ( ± 3.4·10^−6^ cm^2^ V^−1^ s^−1^) and −6.5·10^−5^ cm^2^ V^−1^ s^−1^ ( ± 3.4·10^−6^ cm^2^ V^−1^ s^−1^), while the mean effective mobilities were estimated at −4.2·10^−5^ cm^2^ V^−1^ s^−1^ ( ± 9.7·10^−7^ cm^2^ V^−1^ s^−1^) and −4.6·10^−5^ cm^2^ V^−1^ s^−1^ ( ± 8.6·10^−7^ cm^2^ V^−1^ s^−1^). Using the measured mobilities and Eqs S-7, S-8 and S-9 in section S-2 of the Supplementary Information, a ΔpK_a_ of 3.4·10^−2^ unit was assessed (Table 1).

**Table 1:** pK_a_ values determined for analogs of sialyllactose.

ACD/Lab method	Predicted pK_a_ value
α2,3-sialyllactose	1.9 (±0.7)
α2,6-sialyllactose	2.0 (±0.7)
**Conventional CE method**	**Absolute pK**_**a**_ **value**
α2,3-sialyllactose	1.8 (±0.1)
α2,6-sialyllactose	1.8 (±0.1)
**IS-CE method (** ***N*** ** = 2)**	**Relative pK**_**a**_ **value**
	3.4·10^−2^

This relative ΔpK_a_ therefore reflects a difference in the acidity constants of the two sialylated glycan isomers. Consequently, this led to different electrophoretic mobilities in the working background electrolyte (BGE) (*i*.*e*., 10% AA at pH 2.3) for α2,3- and α2,6-linked sialylated glycopeptides of IgGmAb1 and IVIgG. Considering that other experimental conditions are used in the CE–ESI-MS approach compared to the IS-CE approach (*i*.*e*., longer capillary (90 cm vs 32.5 cm), long-end injection vs short-end injection (89 cm vs 8.5 cm effective length of the capillary) and a higher voltage (5/8 kV vs 20 kV)), a migration time difference of 1 min with MS detection was estimated between the α2,3- and α2,6-linked sialyllactose on the basis of the deduced pK_a_ difference (see Eq. S-7 in section S-2 of the Supplementary Information). These findings supports the result obtained with the co-injection of IgGmAb1 and IVIgG1 illustrated in Fig. 1. The different linkage affects the charge of the sialyllactoses, which may also present a different size, overall impacting the charge-to-size ratio and, thus, the migration behavior. Therefore, the CE-ESI-MS technique appears to be a promising tool for separating sialic acid linkage isomers.

### 2.3 Analysis of prostate specific antigen glycopeptides using CE–ESI-MS

Due to the heterogeneous glycosylation character of PSA, including differentially linked sialic acids, this glycoprotein was selected to investigate the potential of using of CE–ESI-MS for sialic acid linkage isomer differentiation^43^. A state-of-the-art MALDI-TOF-MS platform was also used as orthogonal technique after enzymatic release of the glycans from PSA and an ethyl esterification to distinguish α2,3- from α2,6-linked sialic acids of released glycans, the results were compared to the CE–ESI-MS platform^44^. Moreover, in order to evaluate the migration behavior of the sialylated isomers of PSA, an exosialidase step was performed to allow for the release of α2,3-linked sialic acids from complex glycopeptides.

#### Ethyl esterification

**Figure 2:**
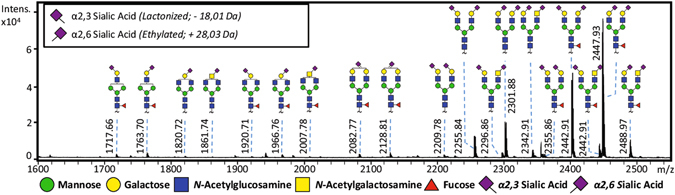
MALDI-TOF-MS spectrum of PNGase F released PSA *N*-glycans treated with ethyl esterification. A total of 37 different glycans were identified (not all data shown, a complete overview can be found in Table S-3, Supplementary Information).

The enzymatically released *N*-glycans from PSA were derivatized using a procedure developed by Reiding *et al*., converting α2,3-linked sialic acids into a lactone (−18 Da) while α2,6-linked sialic acids formed an ethyl ester (+28 Da)^44^. The MALDI-TOF-MS profiling confirmed that the acquired PSA consisted of differentially linked sialic acids (Fig. 2), enabling the identification of 37 glycans (S-6 Supplementary Information, Table S-1). It is worth mentioning that sialic acid attached to a *N*-acetylgalactosamine only revealed an α2,6-linkage, which is supported by literature^10^.

#### CE–ESI-MS(/MS) analysis of a PSA tryptic digest

**Figure 3:**
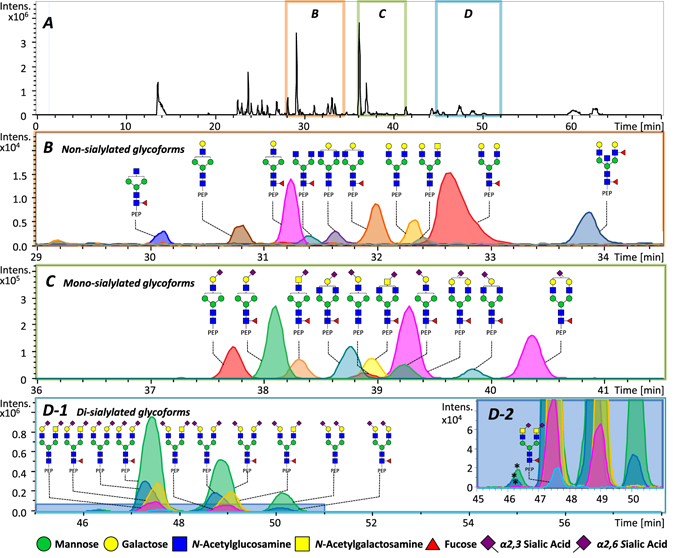
CE–ESI-MS analysis of PSA tryptic (glyco)peptides. (**A**) Representative base peak electropherogram observed for a tryptic digest of PSA. Based on the electrophoretic separation, three distinct clusters were defined. (**B**) Cluster with only non-sialylated glycopeptides of PSA. (**C**) Cluster containing mono-sialylated glycopeptides of PSA. (**D**) Cluster with di-sialylated glycopeptides of PSA. A total of 75 different glycopeptides were identified (not all data shown, a complete overview can be found in Table S-3, Supplementary Information). Peaks with a star (*) were not assigned. The “PEP” label illustrates the tryptic peptide sequence **N**_69_K to which the glycan is attached.

Figure 3 displays the base peak electropherogram and the extracted ion electropherograms (EIEs) obtained for the analysis of a PSA tryptic digest. A relatively high number of glycopeptides (*i*.*e*., 75 glycopeptides, S/N > 9, error < 10 ppm) were identified with the CE–ESI-MS set-up. All glycans were attached to the peptide “**N**_69_K” and are listed in Section S-6 of the Supplementary Information, Table S-1. Notably, a relatively low amount of PSA (*ca*. 1 ng protein weight injected) was necessary to detect these 75 glycopeptides. The base peak electropherogram of a PSA tryptic digest analysis is shown in Fig. 3A while the EIEs of non-sialylated, mono-sialylated, and di-sialylated glycoforms are shown in Fig. 3B–D. Figure 3C and D clearly emphasize that α2,3- and α2,6-linked isomers are baseline resolved, confirming the ability of CE–ESI-MS to discriminate between the different linkages, independently of the glycopeptide structure. The identified compounds are represented in terms of number of hexoses (H), *N*-acetylhexosamines (N), fucoses (F) and sialic acids (S). As illustrated in Fig. S-2 and S-3 of the Supplementary Information, isomer separation was observed for the α2,3- and α2,6-linked sialylated species (H5N4S2, H5N4F1S2, H4N5S2 and H4N5F1S2). Interestingly, a peak with a higher apparent mobility compared to the fully α2,6-di-sialylated species was detected, with a similar isotopic distribution. This peak could not be assigned with MS/MS due to the relatively poor signal intensity. Given the migration in the di-sialylated region of the electropherogram, this unidentified analyte may also be a glycopeptide containing two sialic acids, from which one is an α2,8-linked sialic acid on top of the second sialic acid. However, the presence of a di-sialic acid motif with an α2,8-linkage could not be confirmed with the MALDI-TOF experiments as this α2,8-linked sialic acid is expected to readily form lactones under ethyl esterification conditions, leading to the same *m/z* value as for a glycan with an α2,3-linked sialic acid even though there is a difference in linkage position^45^. Next to that, there are hitherto no internal standards available that would contain this specific linkage. Therefore, similar experiments as performed with the α2,3- and α2,6-linked sialyllactose with IS-CE could not be performed. As a result, further investigations are needed to confirm this hypothesis.

Next to the isomer separation of the differentially linked sialic acids, Fig. 3 also highlights that the glycopeptide separation is highly influenced by the degree of sialylation. This is expected as the migration of the glycopeptide is dependent on the charge-to-size ratio of the analyte in the capillary. Figure 3B illustrates that the non-sialylated glycopeptides migrate first followed by the mono- and di-sialylated species. The sialic acids are expected to carry a negative charge when 10% AA is used as BGE (pH 2.3), resulting in a slower migration in the capillary. More sialic acids on the analyte will therefore increase the migration time. Figure 3B illustrates the EIEs observed for non-sialylated glycopeptides and revealed no isomer separation (co-migration of isomers), indicating that the separation mostly relies on the number of sialic acids attached to the analyte as well as the ΔpK_a_ of the differentially linked sialic acids. Differences in hydrodynamic radii of the analytes appeared to have only a minor influence, as illustrated for glycopeptides with neutral glycans containing 0, 1 or 2 galactose residues which only showed minor differences in migration without differentiation between isomers.

#### CE–ESI-MS analysis after exosialidase treatment

**Figure 4:**
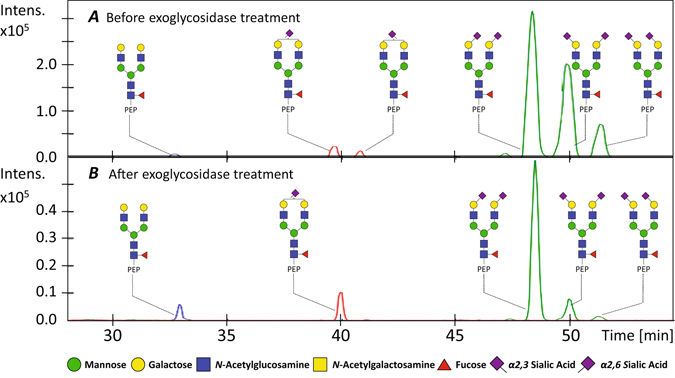
CE–ESI-MS analysis of PSA with and without α2,3-sialidase treatment using CE–ESI-MS after targeted alignment. (**A**) EIEs of the non-sialylated glycopeptide (H5N4F1), the mono-sialylated glycopeptides (H5N4F1S1) and the di-sialylated glycopeptides (H5N4F1S2) of PSA prior to exoglycosidase treatment and (**B**) after exoglycosidase treatment.

Figure 4 shows the EIEs observed for the di-sialylated glycopeptides H5N4F1S2 (blue trace) and H4N5F1S2 (green trace) prior (Fig. 4A) and after (Fig. 4B) treatment with α2,3-sialidase. Both analyses were aligned using the targeted alignment program to ensure that a proper identification could be made of the different isomeric species between the two analyses. The electropherograms obtained for α2,6- and α2,3-sialylated species indicated a significant decrease of the relative abundance of α2,3-sialylated variants. The intensities observed for α2,6-linked mono-sialylated glycopeptide (H5N4F1S1_2,6_) and the non-sialylated glycopeptide (H5N4F1) increased compared to the most abundant peak (α2,6-linked di-sialylated glycopeptide, H5N4F1S2_2,6_) while all α2,3-linked sialylated species (H5N4F1S1_2,3_, H5N4F1S1_2,3_S_2,6_ and H5N4F1S2_2,3_) showed a decreased signal. However, an incomplete reaction was observed for the exoglycosidase treatment which may most likely be attributed to experimental conditions such as (too short) incubation time and (low) amount of enzyme added. Nonetheless, these results confirmed that the CE–ESI-MS set-up enabled a baseline separation of α2,3- and α2,6-sialylated glycopeptides, with α2,3-variants showing a slower migration.

### Synopsis

Little attention has been devoted to the analysis of glycopeptides with CE–ESI-MS so far. This is unfortunate since this technique has shown high separation efficiency and sensitivity in the context of large biomolecules analysis, especially in combination with a sheathless ESI interface^46–49^. For instance, Heemskerk *et al*.^50^ showed that a 40-fold increase in sensitivity with CE–ESI-MS compared to nano-LC–ESI-MS was observed for the analysis of plasma derived IgG1 Fc glycopeptides.

This study presented a novel approach for in-depth and direct analysis of complex glycoproteins with differently linked sialic acids using a CE–ESI-MS platform, without the need for tedious and time-consuming sample preparation^29, 33, 44^. With the developed set-up, 75 different glycopeptides with one single *N*-linked glycosylation site, position R-**N**_69_K-S, were detected for the analysis of a PSA tryptic digest. This CE–ESI-MS platform shows a great potential for PSA glycopeptide analysis with results comparable to an inter-laboratory study performed on PSA and PSA high isoelectric point isoform (bulk-purified proteins based on isoelectric point)^51^. The latter reported that 61 *N*-glycan compositions could be detected using multiple analytical strategies including bottom-up versus top-down analysis, released *N*-glycans analysis by PNGase F, polymeric reversed-phase versus monolith and C8 chromatography, as well as CE.

Nowadays, the concentration of PSA in serum is widely used in clinics for the screening of prostate cancer since elevated PSA serum levels can be indicative of malignancy^52^. However, this screening method shows a poor specificity as various other causes also lead to increased PSA levels in serum^53, 54^. Previous studies indicated that alterations in the glycosylation profile of PSA could provide a more specific tool for diagnosis and prognosis of prostate cancer^43, 55–59^. This study presents a compelling platform for the analysis of these alterations, offering a very competitive tool to improve both specificity and sensitivity. Moreover, it allows to evaluate the PSA glycosylation, a promising approach for early detection, for better prognosis or staging of prostate cancer. However, while currently one ng of protein weight was injected and analyzed, such amounts will be difficult to retrieve from serum of prostate cancer patients, who often have PSA concentrations lower than 10 ng/mL. On the opposite, urine PSA concentrations of prostate cancer patients have been reported with a median value of 425 ng/mL, a sufficient concentration level for this CE–ESI-MS platform^60^. Therefore, the potential of PSA biomarkers derived from urine should be investigated and could open new possibilities for establishing a less invasive but hopefully more specific diagnostic marker than the current clinical PSA test. This has, to the best of our knowledge, not yet been explored. Future perspectives include the analysis of glycoproteins with higher complexity and more glycosylation sites, as well as highly sialylated glycopeptides which may show a negative apparent mobility, rendering their detection critical with the current set-up. Further method development is therefore needed to achieve the analysis of, for instance, tri- or tetra-sialylated glycopeptides.

## Conclusions

This study presents the implementation of a CE–ESI-MS platform enabling the differentiation between α2,3- and α2,6-sialylated glycopeptides without prior derivatization. Where conventional MS(/MS) approaches cannot resolve these isomers due to identical molecular formulas and similar fragmentation patterns, CE enabled the baseline separation of sialylated glycopeptides due to a difference in their electrophoretic mobilities, correlated by a difference in acidity, as shown for the two representative compounds α2,3-sialyllactose and α2,6-sialyllactose. The developed platform was used for the analysis of tryptic PSA glycopeptides, enabling the identification of 75 PSA glycopeptides, a significantly higher amount compared to the 37 PNGase F released *N-*glycan signals detected by MALDI-TOF-MS. This highlights the gain in sensitivity obtained with the developed platform and its potential for biomarker discovery, enabling the possible differentiation between clinically relevant glycopeptides revealing differentially-linked sialic acids.

## Methods

### Materials

Ammonium bicarbonate (ABC), methanol (MeOH) of LC–MS grade and sodium dodecyl sulfate (SDS) were obtained from Merck (Darmstadt, Germany). Glacial acetic acid (AA), DL-diothiotreitol (DTT), iodoacetamide (IAA), 1-hydroxybenzotirazole (HOBt) hydrate and Nonidet P-40 (NP-40) were acquired from Sigma-Aldrich (Steinheim, Germany). Ammonium acetate (AAC), formic acid (FA), trifluoroacetic acid (TFA) and water of LC–MS grade were purchased from Fluka (Steinheim, Germany). Sequencing Grade Modified Porcine Trypsin was obtained from Promega (Madison, WI). Phosphoric acid (H_3_PO_4_), MES (2-(N-morpholino)ethanesulfonic acid), MOPS (3-morpholinopropanesulfonic acid), TRICINE (N-(tris(hydroxymethyl)-methyl)glycine), and CHES (2-(cyclohexylamino)ethane sulfonic acid) were purchased from Fluka (Buchs, Switzerland). Milli-Q water (MQ) was used for the matrix-assisted laser desorption ionization time-of-flight MS (MALDI-TOF-MS) experiments and obtained using a Q-Gard 2 system (Millipore, Amsterdam, Netherlands). HPLC supraGradient acetonitrile (MeCN) originated from Biosolve (Valkenswaard, Netherlands). 1-Ethyl-3-(3-(dimethylamino)propyl)-carbodiimide (EDC) hydrochloride was purchased from Fluorochem (Hadfield, United Kingdom). For the pK_a_ determination, HPLC grade methanol was supplied by Romil (Kölliken, Switzerland), analytical reagent grade acetone by Acros Organics (Basel, Switzerland), hydrochloric acid by Riedel-de-Haën (Buchs, Switzerland), and 1 M Titrinorm sodium hydroxide by VWR (Dietikon, Switzerland). The CEofix^®^ pH 2.5 solutions were obtained from Analis (Namur, Belgium). Recombinant peptide-N-glycosidase F (PNGase F) was acquired from Roche Diagnostics (Mannheim, Germany) and 2.5-dihydroxybenzoic acid (2,5-DHB) from Bruker Daltonics (Bremen, Germany). Lyophilized sialidase S (S*treptococcus pneumoniae*) was obtained from PROzyme (Hayward, CA) and reconstituted in 20 µL MQ prior to use. Recombinant monoclonal IgG1 antibody (IgGmAb1) is derived from Chinese Hamster Ovary (CHO) cells. Human polyclonal IgG (intravenous immunoglobulin; IVIgG) was provided by Sanquin Research (Amsterdam, The Netherlands). Prostate specific antigen (PSA) was acquired from Lee BioSolutions (St. Louis, MO). The two oligosaccharide standards 3′-sialyllactose sodium salt and 6′-sialyllactose sodium salt were obtained from Carbosynth (Compton, United Kingdom).

### Tryptic Digest

A PSA solution (1 µg/µL) was prepared in 25 mM ABC buffer. The reduction of disulphide bridges was carried out at 60 °C for 30 min after addition of DTT (final concentration 2 mM) to the PSA solution. After cooling at room temperature, the sulfide alkylation was performed using IAA (final concentration 6 mM) and the sample was kept in the dark for 30 min. Sulfide alkylation reaction was stopped by addition of DTT (final concentration 6 mM). Finally, porcine trypsin was added to the sample (1:30, *w/w*) followed by overnight digestion at 37 °C.

Tryptic digests of IgGmAb1 and IVIgG solutions were prepared in 25 mM ABC buffer (1 µg/µL) with the addition of porcine trypsin (Promega Madison, WI, enzyme:sample 1:30, *w/w*). Digestion was performed overnight at 37 °C.

### *N*-glycan release

*N*-glycans were released from PSA as described previously^61^. Briefly, 10 µL of 2% SDS was added to 5 µL of PSA (21.5 µg) for denaturation and incubated for 10 min at 60 °C. A mixture (10 µL) consisting out of 4% NP-40, 5x PBS and 1 mU PNGase F (10:10:1, *v/v/v*) was added for the release of the *N*-glycans and incubated overnight at 37 °C.

### Exoglycosidase Treatment

Ten microliters of the digested PSA sample (5.67 µg) was lyophilized and reconstituted in a mixture of 1 µL enzyme Sialidase S, 1 µL of 50 mM sodium acetate (pH 5.5) and 8 µL of MQ, followed by overnight incubation at 37 °C.

### Ethyl esterification

Ethyl esterification was performed as described by Reiding *et al*.^44^. Briefly, the ethylation reagent was a mixture of EDC with HOBt dissolved in EtOH with a final concentration at 0.25 M for both compounds. One microliter of the PNGase F treated PSA was added to 20 µL of ethylation reagent and incubated for 1 h at 37 °C.

### Glycan enrichment by Cotton HILIC SPE

Glycan enrichment was performed using cotton wool hydrophilic interaction liquid chromatography (HILIC) solid phase extraction (SPE) microtips, with a protocol modified from Selman *et al*.^62^. Pipette tips with a volume of 20 µL (Rainin Instrument, Oakland) were filled with approximately 3-mm long cotton thread (Pipoos, Netherlands). This HILIC-SPE sorbent was activated with 20 µL MQ water prior to conditioning with a solution of 85% MeCN (*v/v*), each step being repeated three times. Approximately 0.9 µg of the ethylated PSA glycans was dissolved in 85% MeCN and loaded onto the cotton thread. The ethylated glycans were washed, first with a 20 µL solution of MeCN-MQ-TFA, 85:14:1 (*v/v/v*) followed by 20 µL with a solution of 85% MeCN, both steps were repeated three times. Analytes were eluted with 20 µL of MQ water.

### Determination of pK_a_ values

Details about the theory and comprehensive explanations on the experimental procedure are available in Section S-2 and S-3 of the Supplementary Information.

In order to investigate the possible difference in pK_a_ values for α2,3- and α2,6-sialyllactose, an *in silico* prediction was initially performed as described in S-3.2, Supplementary Information. pK_a_ values were then experimentally determined using CE with ultraviolet (UV) detection. CE-UV experiments were performed with an HP^3D^CE system (Agilent Technologies, Waldbronn, Germany) equipped with an on-capillary diode array detector (8.5 cm from the anode), an autosampler, and a power supply able to deliver up to 30 kV. UV detection was carried out at 195 nm for target compounds and 260 nm for acetone (neutral marker of electro osmotic flow, EOF). CE ChemStation (Agilent Technologies) was used for CE and UV control, data acquisition, and data handling. Analyses were performed with fused silica capillaries (BGB Analytik AG, Böckten, Switzerland) with 50 μm i.d., 32.5 cm total length, and 8.5 cm effective length. The capillaries were coated with a dynamic coating (CEofix®, Analis, Namur, Belgium) to generate a large EOF (*ca*. > 2.2∙10^−4^ cm^2^∙V^−1^∙s^−1^) regardless of the buffer pH. Samples were kept at room temperature in the autosampler and introduced by short-end injection, namely, the polarity was reversed and samples were injected at the detector side. An injection equivalent to 0.4% of the capillary total length was performed by applying a pressure of 12 mbar for 4 s. The capillary was thermostated at 25 °C by a high velocity air stream. Before its first use, the capillary was sequentially rinsed (1 bar) with MeOH, 1 M HCl, water, 1 M NaOH, 0.1 M NaOH, and water (5 min each) prior to the coating procedure. The latter involved capillary rinses with aqueous polymer solutions (CEofix® initiator and accelerator). Between different pH buffers, several washing steps (1 bar) were employed, namely water (1 min), CEofix® initiator (0.5 min), CEofix® accelerator (1 min), and BGE (5 min), followed by pre-electrophoresis (3.5–8 kV, 5 min). Between analyses, the capillary was rinsed (1 bar) with buffer for 1 min. When not in use, the capillary was rinsed with water and stored dry.

For the determination of absolute pK_a_ values, a voltage of 3.5 to 8 kV (depending on the running buffer, see S-6 Supplementary Information, Table S-2) was applied to avoid detrimental effect of Joule heating. Other experimental conditions for the absolute pK_a_ values are described in S-3.3, Supplementary information.

For the relative pK_a_ determination, two phosphate buffers at pH 2.0 and 6.0 were prepared. They were set at a constant ionic strength of 50 mM to avoid variations in effective mobility and keep activity coefficients constant (S-6 Supplementary Information, Table S-3). The buffer pH values were selected to ensure that both forms coexist (*i*.*e*., at pH 2.0) and only the fully deprotonated form exists (*i*.*e*., at pH 6.0). The pH values were measured with a Mettler-Toledo SevenMulti pH meter that was calibrated on a daily basis. The injected sample was a mixture of both investigated compounds at a concentration of 0.1 mg/mL in MQ water and acetone 95:5 (*v/v*). They were injected twice (*N* = 2) at each pH. Voltages of 8 kV and 5 kV were applied when using the BGEs at pH 6.0 and 2.0, respectively.

### MALDI-TOF-MS

MS experiments of ethylated PNGase F released *N*-glycans were performed with MALDI-TOF-MS (UltrafleXtreme, Bruker Daltonics, Bremen, Germany) using FlexControl 3.3 software (Bruker Daltonics) in positive ionization mode. The ethylated PNGase F released *N*-glycans were spotted on an 800-µm AnchorChip plate (Bruker Daltonics) together with 1 µL of 2,5-DHB matrix (5 mg/mL, 1 mM NaOH in MeCN-MQ, 50:50, *v/v*). A peptide calibration standard (Bruker Daltonics) was used for external calibration. FlexAnalysis (Bruker Daltonics) was used for the analysis of the acquired MALDI-TOF-MS data. Modifications of the sialic acids were taken into account (lactonization or ethylation) and all glycan masses were recorded using [M + Na]^+^ ions.

### Capillary electrophoresis – Electrospray ionization – mass spectrometry

All CE experiments were carried out on a CESI 8000 system (SCIEX Separations, Framingham, MA), and 90-cm long bare fused capillaries (i.d. of 30 µm, o.d. of 150 µm) were used. Prior to analysis, the capillary was rinsed thoroughly with 0.1 M NaOH (2.5 min), 0.1 M HCl (2.5 min), water (4 min) and BGE which consisted of 10% AA (*v/v*, pH 2.3). The sample was on-line pre-concentrated using transient isotachophoresis (t-ITP). Prior to injection, ammonium acetate at pH 4.0 (final concentration 100 mM) was added to the sample, which acted as the leading electrolyte. Samples were injected using hydrodynamic injection of 1 psi for 60 s (9 nL, corresponding to 1.4% of the capillary volume). For all experiments, a BGE post plug was added by applying 0.5 psi for 25 s (corresponding to 0.3% of the capillary volume). Separation was carried out by applying a voltage of 20 kV.

The CE system was hyphenated to a UHR-QqTOF maXis Impact HD MS (Bruker Daltonics) *via* a sheathless CE–ESI-MS interface from SCIEX. A capillary voltage of −1300 V was applied to ensure a stable electrospray. All experiments were performed in positive ionization mode. The drying gas (nitrogen) flow rate and temperature were set at 1.5 L/min and 180 °C, respectively. MS data was acquired between *m/z* 200–2200 with a spectral acquisition rate of 1 Hz.

CE–ESI-MS data was analyzed with DataAnalysis 4.2 (Build 387, Bruker Daltonics). Prior to data analysis, all MS spectra were calibrated using sodium adducts detected at the beginning of the electropherogram. The data was manually screened for glycopeptides and compositions were matched based on the exact mass, selectivity and relative intensities (S-6 Supplementary Information, Table S-1). Fragmentation spectra were acquired for 27% of the identified compositional glycopeptide variants of PSA (S-7 Supplementary Information, Fig. S-4–S-7). Extracted ion electropherograms (EIEs, smoothed with a Gaussian fit) were acquired with the first three isotopes of the doubly, triply and quaternary charged analytes using a width of ±0.05 *m/z* unit.

For data alignment, to ensure comparable migration times and reveal the separation of sialic acid linkage isomers, the analysis of IgGmAb1 and IVIgG glycopeptides used 2-aminobenzamide labeled dextran ladder as an internal calibrant. For the alignment of PSA glycopeptides no internal standard was needed as the sample was rich enough to ensure proper alignment without an internal standard. The alignment program is an in-house and open source program, based on the LaCyTools alignment tool^63^. The alignment program is supplied with this manuscript and can be found in S-4 of the Supplementary Information. Briefly, an alignment procedure referred to as “targeted alignment” was achieved by supplying a list (.txt file) of the calibrants with their theoretical *m/z* and expected migration time (t_m_) values. After converting the data file to an mzML file, the algorithm examines the t_m_ and *m/z* regions containing the calibrants. The local maximum within each region is isolated. The t_m_ matching to the local maximum is then coupled to the specified t_m_ of the calibrant. All observed t_m_ and expected t_m_ combinations are then taken as an input array for a second-degree polynomial fit. The function (f) returned by the polynomial fit is used to transform the observed t_m_ of the whole spectrum by performing t_m_(new) = f(t−_m_(old)), resulting in well-aligned spectra. This alignment strategy is opposed to an unbiased alignment^64^, which uses random selection of features and is typically suited for unknown samples that contain a wide range of analytes. Targeted alignment is more adequate for highly purified samples in which the analytes are well known and characterized^63^. Afterwards, the aligned runs were plotted using an in-house developed and open source plotting program, supplied with this manuscript (S-5 of the Supplementary Information).

## Electronic supplementary material

Supplementary Information
