# Supplementary material for: Sialic acid linkage differentiation of glycopeptides using capillary electrophoresis – electrospray ionization – mass spectrometry
Source: Sci Rep. 2017 Jun 16;7:3733. doi: 10.1038/s41598-017-03838-y (PMC5473812; doi:10.1038/s41598-017-03838-y)
Supplement: Supplementary file 1 — Supplementary Information [file 41598_2017_3838_MOESM1_ESM.pdf]

Supplementary Information

Sialic acid linkage differentiation of glycopeptides using capillary electrophoresis – electrospray ionization – mass spectrometry

Guinevere S.M. Kammeijer[1*], Bas C. Jansen[1], Isabelle Kohler[1], Anthonius A.M. Heemskerk[1,2], Oleg A. Mayboroda[1] , Paul J. Hensbergen[1], Julie Schappler[3], Manfred Wuhrer[1]

[1] *Leiden University Medical Center, Center for Proteomics and Metabolomics, Leiden, The Netherlands*

[2] *University of Applied Sciences Leiden, Leiden, The Netherlands*

[3] *University of Geneva, University of Lausanne, School of Pharmaceutical Sciences, Geneva, Switzerland*

[*]**Correspondence**: Guinevere S.M. Kammeijer, Leiden University Medical Center, Center for Proteomics and Metabolomics, P.O. Box 9600, 2300 RC Leiden, The Netherlands; g.s.m.kammeijer@lumc.nl; Tel: +31-71-52-69384

TABLE OF CONTENTS

S-1 SUPPLEMENTARY INFORMATION – RESULTS AND DISCUSSION ii

S-1.1. *In silico* prediction ii

S-1.2. Experimental determination of absolute pk$_a$ values ii

S-2. SUPPLEMENTARY INFORMATION – THEORY iii

S-2.1. Theory behind the experimental determination of the absolute pK$_a$ values by a conventional CE approach iii

S-2.2. Theory behind the experimental determination of the relative pk$_a$ values by internal standard CE (IS-CE) approach v

S-3. SUPPLEMENTARY INFORMATION – EXPERIMENTAL SECTION DETERMINATION OF THE PK$_A$ VALUES OF A-2,3 SIALYLLACTOSE AND A-2,6 SIALYLLACTOSE BY CE vi

S-3.1. Buffers for experimental determination of absolute pk$_a$ values vi

S-3.2. Prediction of pk$_a$ values by an *in silico* approach vi

S-3.3. Experimental determination of absolute pk$_a$ values vi

S-4. SUPPLEMENTARY INFORMATION – ALIGNMENT PROGRAM vii

S-5. SUPPLEMENTARY INFORMATION – EIC EXTRACTOR PROGRAM xi

S-6 SUPPLEMENTARY INFORMATION – TABLES S-1 – S-3 xiv

S-7 SUPPLEMENTARY INFORMATION – FIGURES S-1 – S-10 xviii

S-8 REFERENCES xxviii

S-1 SUPPLEMENTARY INFORMATION – RESULTS AND DISCUSSION

S-1.1. *IN SILICO* PREDICTION

As illustrated in Figure S-8.A, Supporting Information, α2,3-sialyllactose possesses two acidic functions. The predicted pK_a values were 12.0 ± 0.7 (moiety highlighted in green) and 1.9 ± 0.7 (moiety highlighted in yellow). Regarding α2,6-sialyllactose (Figure S-8.B, Supporting Information), the predicted pK_a values of the two acidic functions were 12.0 ± 0.7 (moiety highlighted in green) and 2.0 ± 0.7 (moiety highlighted in yellow). These results suggested that a slight difference may exist between the two isomers in the pK_a values for one of the acidic functions, but this difference is not significant due to the relatively large error associated with the prediction.

S-1.2. EXPERIMENTAL DETERMINATION OF ABSOLUTE pK_a VALUES

As detailed in section S-2 of the Supporting Information, pK_a values can be experimentally determined using CE since the effective mobility (μ_{eff}) of an analyte directly depends on its charge state ($\mu = q/r$) which will differ according to the pH of the solution. By plotting the μ_{eff} measured for both analytes in the different BGEs as a function of pH, a sigmoidal curve is traced, allowing for the practical determination of the pK_a using nonlinear regression.[1] An example of the electropherograms are shown in Figure S-9, Supporting Information for each compound analyzed with the neutral marker at pH 2 and 6. The graphical relationship between μ_{eff} and pH observed for α2,3-sialyllactose and α2,6-sialyllactose are shown in Figure S-10A and S-10B, Supporting Information, an overlay of the two compounds is illustrated in Figure S-10C. The curves of both compounds exhibited two distinct inflection points, namely, between pH 1.5 and 3.5, as well as between pH 10.0 and 12.0, corresponding to two dissociation constants. It is worth mentioning that both compounds presented a difference in effective mobility at certain pH values (*e.g.,* $\Delta\mu_{eff}$ = $4.9{\cdot}10^{-6}$ at pH 2.0 and $\Delta\mu_{eff}$ = $3.9{\cdot}10^{-6}$ at pH 6.0). The pK_a values of both compounds were determined using the appropriate fitting model for diacidic compounds (Eq. S-3). The pK_a values of α2,3-sialyllactose were estimated at 11.7 ± 0.8 and 1.8 ± 0.1. The pK_a values of α2,6-sialyllactose were estimated at 11.6 ± 1.2 and 1.8 ± 0.1. The determined pK_a value of α2,3-sialyllactose and α2,6-sialyllactose expressed an overlapping 95 % confidence interval revealing no statistical differences in the determined pK_a values. However this was to be expected as 95 % confidence intervals are typically higher at the limit of the investigated pH range.

S-2. SUPPLEMENTARY INFORMATION – THEORY

S-2.1. THEORY BEHIND THE EXPERIMENTAL DETERMINATION OF THE ABSOLUTE pK$_a$ VALUES BY A CONVENTIONAL CE APPROACH

The ith dissociation of a fully protonated polyprotic solute H_nX^z is described as follow:

$$H_{n-i+1}X^{z-i+1} \Leftrightarrow H_{n-i}X^{z-i} + H^+ \qquad \text{(S-1)}$$

Where n is the total number of ionizable groups and z the charge of the fully protonated species. For measurements performed in non-ideal solutions, the thermodynamic dissociation constant of the ith dissociation step is defined as:

$$K_{ai}(thermodynamic) = \frac{[H_{n-i}X^{z-i}]}{[H_{n-i+1}X^{z-i+1}]} \cdot \frac{\gamma^{z-i}}{\gamma^{z-i+1}} \cdot a_{H^+} \qquad \text{(S-2)}$$

Where a_{H^+} is the proton activity, terms in brackets represent molar concentrations of the electrical species of the solute and γ terms are the activity coefficients of charged species other than proton.

The effective mobility (μ_{eff}) of a polyprotic compound H_nX^z, coexisting in different ionized states at a given pH, depends on the molar fraction (χ_j) and on the effective mobility of each individual species:

$$\mu_{eff} = \sum_{i=0}^{n} \chi_{H_{n-i}X^{z-i}} \cdot \mu_{H_{n-i}X^{z-i}} \qquad \text{(S-3)}$$

Therefore,

$$\mu_{eff} = \sum_{i=0}^{n} \frac{[H_{n-i}X^{z-i}]}{\sum_{i=0}^{n}[H_{n-i}X^{z-i}]} \cdot \mu_{H_{n-i}X^{z-i}} \qquad \text{(S-4)}$$

Using Eq. S-2, the previous equation can be rewritten as:

$$\mu_{eff} = \sum_{i=0}^{n} \frac{\left[\prod_{j=1}^{i} K_{aj}\right] \cdot a_{H^+}^{n-i}}{\sum_{i=0}^{n}\left[\prod_{j=1}^{i} K_{aj}\right] \cdot a_{H^+}^{n-i}} \cdot \mu_{H_{n-i}X^{z-i}} \qquad \text{(S-5)}$$

Considering $K_{aj} = 10^{-pK_{aj}}$ and $a_{H^+}^{n} = 10^{-npH}$, Eq. S-5 can be rearranged as a function of pH and $-pK_{aj}$:

$$\mu_{eff} = \sum_{i=0}^{n} \frac{\left[\prod_{j=1}^{i} 10^{-pK_{aj}}\right] \cdot 10^{(i-n)pH}}{\sum_{i=0}^{n}\left[\prod_{j=1}^{i} 10^{-pK_{aj}}\right] \cdot 10^{(i-n)pH}} \cdot \mu_{H_{n-i}X^{z-i}} \qquad \text{(S-6)}$$

This equation allows the determination of pK_a values from a plot of μ_{eff} as a function of pH.
Practically, μ_{eff} $(cm^2 \cdot V^{-1} \cdot s^{-1})$ of an analyte can be measured as:

$$\mu_{eff} = \frac{L_{eff} \cdot L_{tot}}{U} \cdot \left(\frac{1}{t_m} - \frac{1}{t_{EOF}}\right) \qquad \text{(S-7)}$$

Where, t_m is the migration time of the analyte, t_{EOF} the migration time of acetone, used as the EOF marker. U is the applied voltage (V), L_{tot} the total capillary length (cm) and L_{eff} the effective capillary length (cm).

S-2.2. THEORY BEHIND THE EXPERIMENTAL DETERMINATION OF THE RELATIVE pK_a VALUES BY INTERNAL STANDARD CE (IS-CE) APPROACH

Considering both analogs, two different expressions for Eq. S-6 can be defined, one for the first analyte (α2,3) and the other for the other analyte (α2,6). If both equations are subtracted and rearranged, Eq. S-8 is obtained:

$$\Delta pKa = pK_{a_{\alpha 2,3}} - pK_{a_{\alpha 2,6}} = logQ_{\alpha 2,3} - logQ_{\alpha 2,6} \qquad \text{(S-8)}$$

Where Q is a quotient that involves the limiting mobility (i.e., of the fully deprotonated form, μ_{A^-} ¯, obtained at pH 6.0) of the acid and its effective mobility (i.e., the mobility at a pH where the ionized and the neutral form of the acid coexist, μ_{eff}, obtained at pH 2.0):

$$Q = \frac{\mu_{A^-} - \mu_{eff}}{\mu_{eff}} \qquad \text{(S-9)}$$

The mobility values are directly calculated from the migration times of the analyte and the EOF marker, t_m and t_{EOF}, respectively, by Eq. S-7.

As illustrated in Eq. S-8, the pH term is absent indicating that the equation is therefore also pH independent. Since, both Eq. S-8 and S-9 are pH independent, the limiting and effective mobilities of both analytes are the only parameters to be determined (Eq. S-9).[2] This means that both compounds have to be injected together in a buffer at a pH where they are totally ionized in order to determine μ_{A^-}, and in a buffer at a pH where they are only partially ionized to obtain the μ_{eff}., which was obtained at pH 6 and 2, respectively. For both cases, a phosphate buffer at the same ionic strength was used (Table S-2, Supporting Information). As mentioned above, the pH of the buffer was less critical than with the conventional approach since the pH values of both buffers do not need to be precisely measured.

S-3. SUPPLEMENTARY INFORMATION – EXPERIMENTAL SECTION DETERMINATION OF THE pK$_a$ VALUES OF α-2,3 SIALYLLACTOSE AND α-2,6 SIALYLLACTOSE BY CE

S-3.1. BUFFERS FOR EXPERIMENTAL DETERMINATION OF ABSOLUTE pK$_a$ VALUES

Twenty-two buffers covering a pH range from pH 1.5 to 12.0 were prepared. All buffers were set at a constant ionic strength of 50 mM to avoid variations in effective mobility and keep the activity coefficients constant (Table S-1, Supporting Information). The nature of the buffers was carefully selected to ensure the best buffer capacity as well as minimal interactions with the analytes. The pH values were measured with a Mettler-Toledo SevenMulti pH meter (Schwerzenbach, Switzerland), daily calibrated with four aqueous solutions at pH 2.00, 4.00, 7.00, and 10.00 from Riedel-de-Haën (Buchs, Switzerland). Samples were set at a concentration of 0.5 mg/mL in MQ water and acetone 95:5 (*v/v*). Acetone was used as a marker of the electroosmotic flow (EOF).

S-3.2. PREDICTION OF pK$_a$ VALUES BY AN *IN SILICO* APPROACH

Prediction of the pK$_a$ values of α2,3-sialyllactose and α2,6-sialyllactose was performed with ACD/Labs software version 12.01 (Advanced Chemistry Development, Inc., Toronto, ON, Canada). The prediction did not include the influence of the intramolecular hydrogen bonds which may impact the estimated pK$_a$ values. Estimation of the error associated to the predicted values was also reported.

S-3.3. EXPERIMENTAL DETERMINATION OF ABSOLUTE pK$_a$ VALUES

Absolute pK$_a$ values were determined using a procedure developed by Geiser *et al.*[1]. Briefly, each sample was injected once at each pH. The effective mobility (μ_{eff}) was calculated from the migration times of the analyte and the neutral marker using Eq. S-7.

Calculated effective mobilities were reported as a function of pH, giving rise to sigmoidal curves. With a GraphPad Prism 6.01 software (GraphPad Software, San Diego, CA, USA), non-linear regressions were performed to determine apparent pK$_a$ values at I=50 mM with Eq. S-10 used for diacidic compounds $(N = 2, z = 0)$.

$$\mu_{eff} = \frac{\mu_{HX^-} 10^{-pK_{a1}-pH} + \mu_{X^{2-}} 10^{-pK_{a2}-pK_{a1}}}{10^{-2pH}+10^{-pK_{a1}-pH}+10^{-pK_{a2}-pK_{a1}}} \quad \text{(S-10)}$$

S-4. SUPPLEMENTARY INFORMATION – ALIGNMENT PROGRAM

For the use of the alignment program a mzML file needs to be created of the generated MS file. Furthermore an alignment file (.txt) needs to be provided that holds the information regarding which *m/z* feature needs to be used and the desired migration time (t_m). While running the program the time tolerance should be specified (sec) as well the *m/z* tolerance.

```
#! /usr/bin/env python
import base64
import glob
import matplotlib.pyplot as plt
import numpy
from scipy.interpolate import splrep
import struct
import sys
import zlib

def decoder(string,precision,compressed):
    """ processes a string and returns an array.
    """
        if not string:
            return []
        if precision == 64:
            precision = 'd'
        else:
            precision = 'f'
        data = base64.b64decode(string)
        if compressed == 1:
            data = zlib.decompress(data)
        count = len(data) / struct.calcsize('<' + precision)
        result = struct.unpack('<' + precision * count, data[0:len(data)])
        return result

def feature_reader(file):
    """ reads the contents of the 'features.txt' file and stores
    the relevant values in a list.
    """
    features = []
    with open(file,'r') as fr:
        for line in fr:
            if line[0][0].isdigit():
                line = line.rstrip().split()
                features.append(line)
            else:
                continue
    return features

def targeted_mzML_reader(file,targetMass,targetTime,massTol,timeTol):
    """ reads the contents of an input mzML. The function also
    hands chunks over to the processing function.
    """
```

```
with open(file,'r') as fr:
    # switch to keep track if we are at m/z or intensity binary data
    switch = 0
    peakIntensity = 0
    peakTime = 0
    # default value for decoding/uncompress
    compression = 0
    precision = 32
    for line in fr:
        if 'zlib compression' in line:
            compression = 1
        if '64-bit' in line:
            precision = 64
        if '<spectrumList count=\"' in line:
            number = line.split(" ")
            for x in number:
                if 'count' in x:
                    number = x.split("\"")[1]
        if 'scan start time' in line:
            time = line.split(" ")
            for x in time:
                if 'value' in x:
                    time = x.split("\"")[1]
        if '<spectrum index=\"' in line:
            index = line.split(" ")
            for x in index:
                if 'index' in x:
                    index = x.split("\"")[1]
        if 'ms level' in line:
            level = line.split(" ")
            for x in level:
                if 'value' in x:
                    level = x.split("\"")[1]
        if 'isolation window target m/z' in line and int(level) == 2:
            precursor = line.split(" ")
            for x in precursor:
                if 'value' in x:
                    precursor = x.split("\"")[1]
        if '<binary>' in line:
            if float(time) > float(targetTime) - timeTol and float(time) < float(targetTime) + timeTol:
                if switch == 0:
                    mz_array = line.split("<binary>")[1]
                    mz_array = mz_array.split("<binary>")[0]
                    mz_array = decoder(mz_array,precision,compression)
                    #precision = 32 # Reset to default precision [6-2-2014]
                    switch = 1
                elif switch == 1:
                    int_array = line.split("<binary>")[1]
                    int_array = int_array.split("<binary>")[0]
                    int_array = decoder(int_array,precision,compression)
                    switch = 0
```

```
                        data = zip(mz_array,int_array)
                        if feature_finder(data,float(targetMass)- massTol,float(targetMass)+massTol)
                    > peakIntensity:peakIntensity = feature_finder(data,float(targetMass)-
                            massTol,float(targetMass)+massTol)
                            peakTime = time
    return (peakTime,peakIntensity)

def feature_finder(data,lowMass,highMass):
    """Reads the current spectrum and returns the maximum
    intensity of searched feature"""
    intensity = 0
    for i in data:
        if i[0] > lowMass and i[0] < highMass:
            if i[1] > intensity:
                intensity = i[1]
        return intensity

def pair_plot(data,file):
    """Plots the set of timepoints (expected and observed) to
    visualize how well the alignment would fit.
    """
    output = file.split(",")[0]+".png"
    expected = []
    observed = []
    for i in data:
        expected.append(i[0])
        observed.append(i[1])
    # The following lines are for the spline (once I figure it out)
    # The following lines are for the Numpy.Polyfit
    z = numpy.polyfit(observed,expected,2)
    f = numpy.poly1d(z)
    x_new = numpy.linspace(expected[0],expected[-1],50)
    y_new = f(x_new)
    plt.figure()
    plt.scatter(expected,observed,marker='o')
    plt.plot(x_new,y_new,color='red')
    plt.xlabel('Expected RT')
    plt.ylabel('Observed RT')
    plt.savefig(output)
    #plt.show()
    return f

def transform_mzML(file,polynomial):
    """Reads the mzML file and transforms the reported retention
    time by the specified polynomial function.
    """
    with open (file,'r') as fr:
        output = "aligned_"+str(file)
        print "Writing output file: "+output
        with open(output,'w') as fw:
            for line in fr:
```

```
                if 'scan start time' in line:
                    time = line.split(" ")
                    # Get 'current' time
                    for x in time:
                        if 'value' in x:
                            time = x.split("\"")[1]
                    # Calculate 'aligned' time
                    newTime = str(polynomial(float(time)))
                    #if float(time) > 2250.0 and float(time) < 2300.0:
                        #print time, newTime
                    line = line.replace(time,newTime)
                    fw.write(line)
                else:
                    fw.write(line)
def main():
    timeTol = float(raw_input("Please specify the time tolerance in seconds?\n"))
    massTol = float(raw_input("Please specify the m/z tolerance?\n"))
    features = feature_reader('features.txt')
    for file in glob.glob("*.mzML"):
        timePairs = []
        print "Processing file: "+str(file)
        retTime = 0
        for i in features:
            print "- Feature: "+str(i)
            results = targeted_mzML_reader(file,i[0],i[1],massTol,timeTol)
            #print results # Enable this line for 'logging' purposes
            timePairs.append((float(i[1]),float(results[0])))
        polynomial = pair_plot(timePairs,file)
        transform_mzML(file,polynomial)

if __name__ == "__main__":
    """ Main program
    """
    main()
```

S-5. SUPPLEMENTARY INFORMATION – EIC EXTRACTOR PROGRAM

After usage of the alignment program the generated mzXML file can be used to extract ion electropherograms. While running the program the targeted *m/z* value needs to be specified and a corresponding color (b,g,r,c,m,y,k,w). After targeting all the desired *m/z* values, the *m/z* tolerance can be specified.

```
#! /usr/bin/env python
from matplotlib import font_manager
from scipy.optimize import curve_fit
import glob
import base64
import math
import matplotlib
import matplotlib.pyplot as plt
import numpy
import sys
import struct
import zlib

def decoder(string,precision,compressed):
    """ processes a string and returns an array.
    """
        if not string:
            return []
        if precision == 64:
            precision = 'd'
        else:
            precision = 'f'
        data = base64.b64decode(string)
        if compressed == 1:
            data = zlib.decompress(data)
        count = len(data) / struct.calcsize('<' + precision)
        result = struct.unpack('<' + precision * count, data[0:len(data)])
        return result

def mzML_reader(file,target,color,error):
        """ reads the contents of an input mzML.
        """
        points=[]
        with open(file,'r') as fr:
            num_ms1 = 0
            switch = 0
            # default value for decoding/uncompress
            compression = 0
            precision = 32
            for line in fr:
                # The following 2 'if statements' might be optimized
                if 'zlib compression' in line:
                    compression = 1
                if '64-bit' in line:
```

```
                    precision = 64
                if '<spectrumList count=\"' in line:
                    number = line.split(" ")
                    for x in number:
                        if 'count' in x:
                            number = x.split("\"")[1]
                if 'scan start time' in line:
                    time = line.split(" ")
                    for x in time:
                        if 'value' in x:
                            time = x.split("\"")[1]
                if '<spectrum index=\"' in line:
                    index = line.split(" ")
                    for x in index:
                        if 'index' in x:
                            index = x.split("\"")[1]
                            # Simple progress indicator
                            if int(index) % 100 == 0:
                                print "Spectrum: "+index+" of total "+str(number)
                if 'ms level' in line:
                    level = line.split(" ")
                    for x in level:
                        if 'value' in x:
                            level = x.split("\"")[1]
                if 'isolation window target m/z' in line and int(level) == 2:
                    precursor = line.split(" ")
                    for x in precursor:
                        if 'value' in x:
                            precursor = x.split("\"")[1]
                if '<binary>' in line:
                    if switch == 0:
                        mz_array = line.split("<binary>")[1]
                        mz_array = mz_array.split("</binary>")[0]
                        mz_array = decoder(mz_array,precision,compression)
                        switch = 1
                    elif switch == 1:
                        int_array = line.split("<binary>")[1]
                        int_array = int_array.split("</binary>")[0]
                        int_array = decoder(int_array,precision,compression)
                        switch = 0
                if '</spectrum>' in line:
                    data = zip(mz_array,int_array)
                    if int(level) == 1:
                        for peak in data:
                            if peak[0] > target-error and peak[0] < target+error:
                                points.append((file,target,color,time,peak[1]))
    return points
def gaussFunction(x, *p):
    A, mu, sigma = p
    return A*numpy.exp(-(x-mu)**2/(2.*sigma**2))
def plotter(runs):
```

```
        # Font parameters
        font = {'family' : 'calibri',
                'size'   : '26'}
    matplotlib.rc('font',**font)
    # Determines the maximum intensity to get the order of magnitude
    maxIntensity = 0
    for i in runs:
        int_array = []
        for j in i:
            if abs(float(j[4])) > maxIntensity:
                maxIntensity = abs(float(j[4]))
    orderMagnitude = math.floor(math.log(maxIntensity,10))
    # Plot the data that was given to this function
    for i in runs:
        time_array = []
        int_array = []
        for j in i:
            time_array.append(abs(float(j[3]))/60)
            int_array.append(abs(float(j[4]))/math.pow(10,orderMagnitude))
        y_av = movingaverage(int_array,10)
        #dataLabel = i[0][0].decode("utf-8").split(".")[0]+" - "+str(i[0][1])
        #plt.plot(time_array,y_av,label=dataLabel,color=i[0][2])
        plt.plot(time_array,y_av,linewidth=1.0,color=i[0][2])
        plt.xlabel('Time (min)')
        plt.ylabel('Intensity (10${\mathrm{^'+str(int(orderMagnitude))+'}}$)')
        plt.xlim(30,55)
    # plt.legend(loc=2)
    plt.show()
def movingaverage(interval, window_size):
    window = numpy.ones(int(window_size))/float(window_size)
    return numpy.convolve(interval, window, 'same')
def main():
    runs = []
    targets = []
    target = 1
    while target != "":
        target = raw_input("Please enter target m/z or hit enter to stop\n")
        color = raw_input("Please select a color for this target (ie: b,g,r,c,m,y,k,w)\n")
        if target != "":
            targets.append((float(target),str(color)))
    print "Selected peaks are: "+str(targets)
    error = float(raw_input("Please enter tolerance\n"))
    for file in glob.glob("*.mzML"):
        for target in targets:
            runs.append(mzML_reader(file,target[0],target[1],error))
    plotter(runs)

if __name__ == "__main__":
    """ Main program
    """
    main()
```

S-6 SUPPLEMENTARY INFORMATION – TABLES S-1 – S-3

Table S-1. Theoretical and observed glycopeptide and glycan mass during the CE–ESI-MS and MALDI-TOF-MS analysis of PSA with the corresponding deviation and ppm error (<10). The "PEP" label illustrates the tryptic peptide sequence $N_{69}K$ to which the glycan is attached.

			CESI-MS									MALDI-TOF-MS			
Glycan Species			[M+2H]$^{2+}$	Observed	deviation	PPM error	[M+3H]$^{3+}$	Observed	deviation	PPM error	MS2*	[M+Na]$^{+}$	Observed	deviation	PPM error
	Non-sialylated/ Non-sulphated														
1	H3N3F1	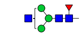	751.809	751.810	0.001	1.08	501.542	ND				1282.45	ND		
2	H4N3	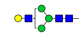	759.807	759.806	-0.001	-1.77	506.874	ND				1298.45	ND		
3	H3N4	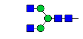	780.320	780.321	0.001	1.00	520.549	ND				1339.48	ND		
4	H4N3F1	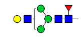	832.836	832.835	-0.001	-0.96	555.560	ND				1444.51	ND		
5	H3N4F1	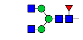	853.349	853.348	-0.001	-1.61	569.235	ND				1485.53	ND		
6	H4N4	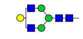	861.347	861.346	-0.001	-1.08	574.567	ND				1501.53	ND		
7	H4N4F1	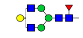	934.375	934.375	-0.001	-0.63	623.253	ND				1647.59	ND		
8	H5N4	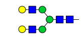	942.373	942.372	-0.001	-1.43	628.585	ND				1663.58	ND		
9	H4N5	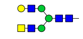	962.886	962.887	0.001	0.71	642.260	ND				1704.61	ND		
10	H5N4F1	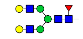	1015.402	1015.401	0.000	-0.49	677.271	ND				1809.64	1809.64	0.00	2.21
11	H5N5F2	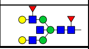	1189.971	1189.971	0.001	0.64	793.650	793.652	0.002	3.06		2158.78	ND		
	Monosialylated/ Sulphated														
12	H3N4F1 [SO$_3$]$^-$	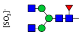	893.327	893.326	-0.001	-1.01	595.888	ND				1565.53	ND		
13	H4N3S$_{2,3}$1	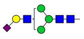	905.355	905.353	-0.001	-1.50	603.906	ND				1571.53	1571.51	-0.02	-15.27
14	H4N3S$_{2,6}$1	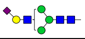	905.355	905.354	-0.001	-0.94	603.906	ND			X	1617.58	1617.56	-0.02	-10.51
15	H3N5 [SO$_3$]$^-$	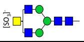	921.838	921.837	-0.001	-0.98	614.895	ND				1622.51	ND		
16	H3N4S$_{2,6}$1	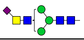	925.868	925.867	-0.001	-1.33	617.581	ND			X	1658.60	1658.60	0.00	-0.60
17	H4N3F1S$_{2,3}$1	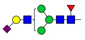	978.384	978.382	-0.001	-1.44	652.592	ND			X	1717.59	1717.59	0.00	0.00
18	H4N3F1S$_{2,6}$1	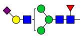	978.384	978.382	-0.001	-1.34	652.592	ND			X	1763.63	1763.64	0.00	2.84
19	H5N3S$_{2,3}$1	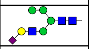	986.381	986.381	0.000	-0.47	657.923	ND				1733.59	ND		
20	H5N3S$_{2,6}$1	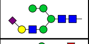	986.381	986.381	0.000	-0.27	657.923	ND				1779.63	1779.64	0.01	3.93
21	H3N4F1S$_{2,6}$1	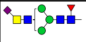	998.897	998.895	-0.002	-1.78	666.267	ND			X	1804.66	1804.66	0.00	0.55
22	H4N4S$_{2,3}$1	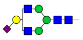	1006.894	1006.893	-0.001	-1.43	671.599	671.598	-0.001	-1.20		1774.61	1774.62	0.01	3.38
23	H4N4S$_{2,6}$1	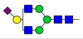	1006.894	1006.893	-0.001	-0.83	671.599	671.596	-0.003	-4.18	X	1820.66	1820.65	0.00	-1.10
24	H3N5S$_{2,6}$1	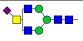	1027.408	1027.406	-0.001	-1.28	685.274	685.272	-0.003	-4.12	X	1861.68	1861.69	0.01	3.76
25	H5N3F1S$_{2,3}$1	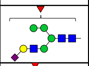	1059.410	1059.413	0.003	3.00	706.609	ND				1879.65	ND		
26	H5N3F1S$_{2,6}$1	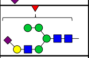	1059.410	1059.413	0.003	3.00	706.609	ND				1925.69	ND		
27	H4N5F1 [SO$_3$]$^-$	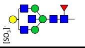	1075.894	1075.891	-0.003	-2.79	717.598	ND				1930.62	ND		
28	H4N4F1S$_{2,3}$1	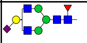	1079.923	1079.922	-0.001	-1.38	720.285	720.284	-0.001	-0.80		1920.67	1920.66	-0.01	-7.29
29	H4N4F1S$_{2,6}$1	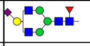	1079.923	1079.922	-0.001	-1.11	720.285	720.284	-0.001	-1.36	X	1966.71	1966.71	0.00	-0.51
30	H5N4S$_{2,3}$1	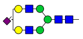	1087.921	1087.919	-0.002	-1.52	725.616	725.615	-0.001	-1.54		1936.67	1936.65	-0.01	-6.71
31	H5N4S$_{2,6}$1	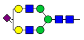	1087.921	1087.919	-0.001	-1.15	725.616	725.615	-0.001	-1.54	X	1982.71	1982.71	0.00	1.97
32	H3N5F1S$_{2,6}$1	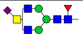	1100.436	1100.435	-0.002	-1.52	733.960	733.959	-0.001	-1.90	X	2007.74	2007.73	-0.01	-3.49
33	H4N5S$_{2,3}$1	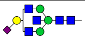	1108.434	1108.433	-0.001	-0.93	739.292	739.291	-0.001	-0.85		1977.69	ND		
34	H4N5S$_{2,6}$1	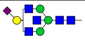	1108.434	1108.433	-0.001	-0.57	739.292	ND				2023.74	2023.73	-0.01	-3.95
35	H3N6S$_{2,6}$1	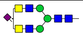	1128.947	1128.946	-0.001	-0.71	752.967	752.967	-0.001	-1.26		2064.76	2064.76	-0.01	-2.42

36	$H6N3F1S_{2,3}1$	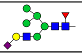	1140.436	1140.434	-0.002	-1.96	760.627	760.627	0.000	0.57		2041.70	ND		
37	$H6N3F1S_{2,6}1$	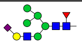	1140.436	1140.437	0.000	0.15	760.627	ND				2087.74	ND		
38	$H5N4F1S_{2,3}1$	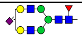	1160.950	1160.948	-0.002	-1.73	774.302	774.301	-0.001	-1.27	X	2082.72	2082.72	0.00	-1.44
39	$H5N4F1S_{2,6}1$	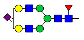	1160.950	1160.948	-0.002	-1.47	774.302	774.301	-0.001	-1.53	X	2128.77	2128.77	0.00	0.47
40	$H4N5F1S_{2,3}1$	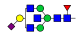	1181.463	1181.461	-0.002	-1.93	787.978	787.976	-0.002	-2.03		2123.75	2123.74	-0.01	-3.77
41	$H4N5F1S_{2,6}1$	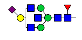	1181.463	1181.461	-0.002	-1.42	787.978	787.972	-0.006	-7.36		2169.79	2169.78	-0.02	-6.91
42	$H3N6F1S_{2,6}1$	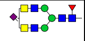	1201.976	1201.974	-0.002	-1.54	801.653	801.651	-0.002	-2.77		2210.82	ND		
43	$H5N4F2S_{2,3}1$	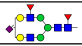	1233.979	1233.978	-0.001	-0.86	822.988	822.987	-0.002	-1.89		2228.78	ND		
44	$H5N4F2S_{2,6}1$	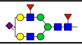	1233.979	1233.977	-0.002	-1.26	822.988	822.988	-0.001	-1.04		2274.82	ND		
45	$H4N5F2S_{2,3}1$	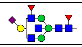	1254.492	1254.490	-0.002	-1.22	836.664	836.662	-0.002	-2.35		2269.81	ND		
46	$H4N5F2S_{2,6}1$	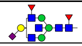	1254.492	1254.491	-0.001	-1.06	836.664	836.663	-0.001	-0.80		2315.85	ND		
47	$H5N5F1S_{2,3}1$	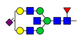	1262.489	1262.488	-0.002	-1.34	841.996	841.995	0.000	-0.48		2285.80	ND		
48	$H5N5F1S_{2,6}1$	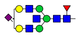	1262.489	1262.490	0.001	0.48	841.996	841.995	-0.001	-0.84		2331.85	ND		
49	$H3N6F2S_{2,6}1$	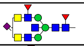	1275.005	1275.004	-0.001	-0.79	850.339	ND				2356.88	ND		
50	$H5N5F2S_{2,3}1$	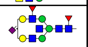	1335.518	1335.517	-0.002	-1.16	890.681	890.681	-0.001	-1.10		2431.86	ND		
51	$H5N5F2S_{2,6}1$	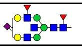	1335.518	1335.517	-0.001	-1.08	890.681	890.681	-0.001	-1.10		2477.90	ND		
	**Di-sialylated/ Mono-sialylated with a sulphate group**														
52	$H4N5F1S_{2,3}1[SO_3]^-$	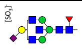	1221.441	1221.441	0.000	0.00	814.630	814.630	0.000	0.37		1930.62			
53	$H4N5F1S_{2,6}1[SO_3]^-$	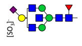	1221.441	1221.439	-0.002	-1.64	814.630	814.629	-0.001	-1.23		2249.75			
54	$H5N4S_{2,3}2$	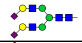	1233.468	1233.467	-0.001	-1.02	822.648	822.647	-0.001	-1.36		2209.75	2209.74	-0.01	-6.34
55	$H5N4S_{2,3}1S_{2,6}1$	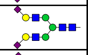	1233.468	1233.466	-0.002	-1.91	822.648	822.647	-0.001	-1.73	X	2255.79	2255.79	-0.01	-2.66
56	$H5N4S_{2,6}2$	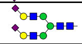	1233.468	1233.466	-0.002	-1.91	822.648	822.647	-0.001	-1.48	X	2301.83	2301.84	0.00	0.96
57	$H4N5S_{2,3}1S_{2,6}1$	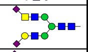	1253.982	1253.980	-0.002	-1.54	836.324	836.323	-0.001	-1.48		2296.82	2296.81	-0.01	-3.27
58	$H4N5S_{2,6}2$	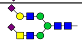	1253.982	1253.980	-0.002	-1.22	836.324	836.322	-0.001	-1.60	X	2342.86	2342.86	0.00	-0.60
59	$H3N6S_{2,6}2$	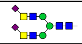	1274.495	1274.491	-0.004	-3.38	849.999	849.998	-0.001	-1.24		2383.89	2383.87	-0.02	-7.55
60	$H5N4F1S_{2,3}2$	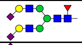	1306.497	1306.495	-0.003	-2.08	871.334	871.333	-0.001	-1.59	X	2447.89	2447.89	0.00	-1.51
61	$H5N4F1S_{2,3}1S_{2,6}1$	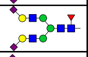	1306.497	1306.494	-0.003	-2.61	871.334	871.333	-0.001	-1.02	X	2401.85	2401.86	0.00	1.67
62	$H5N4F1S_{2,6}2$	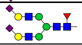	1306.497	1306.495	-0.002	-1.69	871.334	871.333	-0.001	-1.48	X	2355.81	2355.81	0.00	1.27
-	$H5N5S_{2,3}2$	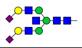	1335.008	ND			890.341	ND				2412.83	ND		
-	$H5N5S_{2,3}1S_{2,6}1$	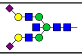	1335.008	ND			890.341	ND				2458.87	ND		
63	$H5N5S_{2,6}2$	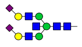	1335.008	1335.016	0.008	5.99	890.341	890.340	-0.001	-1.24		2504.91	ND		
64	$H4N5F1S_{2,6}2$	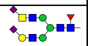	1327.011	1327.007	-0.003	-2.63	885.010	885.008	-0.001	-1.59	X	2488.91	2488.92	0.01	4.02
65	$H4N5F1S_{2,3}1S_{2,6}1$	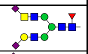	1327.011	1327.008	-0.003	-2.10	885.010	885.008	-0.002	-1.93	X	2442.88	2442.87	-0.01	-2.46
66	$H3N6F1S_{2,6}2$	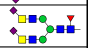	1347.524	1347.516	-0.008	-5.98	898.685	898.682	-0.003	-3.14		2529.95	2529.94	-0.01	-2.77
67	$H6N5F1S_{2,3}2$	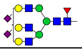	1489.063	ND			993.045	993.044	-0.001	-1.31		2720.94	2720.95	0.01	4.04
68	$H6N5F1S_{2,3}1S_{2,6}1$	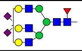	1489.063	ND			993.045	993.043	-0.002	-1.81		2766.98	2766.98	-0.01	-2.53
69	$H6N5F1S_{2,6}2$	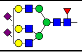	1489.063	ND			993.045	993.045	0.000	0.00		2813.03	2813.03	0.01	2.84

	Tri-sialylated															
70	H6N5$S_{2,3}$3	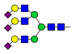	1561.582	1561.582	0.000	-0.04	1041.391	1041.391	0.001	0.62		2986.09	ND			
71	H6N5$S_{2,3}$1(2)$S_{2,6}$2(1)	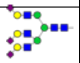	1561.582	1561.593	0.011	7.19	1041.391	1041.391	0.000	0.42		2894.01	ND			
72	H6N5S2,31(1)S2,62(2)	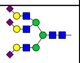	1561.582	ND			1041.391	1041.389	-0.002	-2.17		2940.05	ND			
-	H6N5$S_{2,6}$3	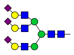	1561.582	ND			1041.391	ND				2847.97	ND			
73	H6N5F1$S_{2,3}$3	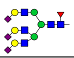	1634.611	1634.618	0.006	3.90	1090.077	1090.077	0.000	-0.02		3132.15	ND			
74	H6N5F1$S_{2,3}$2(1)$S_{2,6}$1(2)	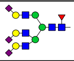	1634.611	1634.620	0.009	5.68	1090.077	1090.076	-0.001	-0.58		3040.07	ND			
75	H6N5F1$S_{2,3}$2(2)$S_{2,6}$1(1)	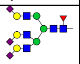	1634.611	1634.622	0.011	6.84	1090.077	1090.076	-0.001	-0.58		3086.11	ND			
-	H6N5F1$S_{2,6}$3	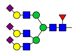	1634.611	ND			1090.077	ND				2994.03	ND			

* Glycan compositions are given in terms of number of hexoses (H), N-acetylglucosamines (N), fucoses (F) and sialic acids (S).

X confirmed with MS2 and ND is not detected

Selected mass is used for the MS2 confirmation

Table S-2. Preparation of buffers at 50 mM ionic strength and applied voltages for the conventional CE method. The preparation of the buffers can be found in the article by Henchoz *et al.*[3]

Buffer composition		Theoretical pH	Measured pH	Buffer capacity	Applied voltage
Buffered species (mM)	Buffering species (mM)	(25°C)	(25°C)	(mM / pH unit)	(kV)
H_3PO_4 (220.5)	NaOH (11)	1.5	1.495	178.6	3.5
H_3PO_4 (103.9)	NaOH (37.7)	2.0	1.988	89.3	5.0
H_3PO_4 (67.0)	NaOH (46.1)	2.5	2.502	38.4	6.0
HCOOH (278.9)	NaOH (48.8)	3.0	2.965	105.1	6.0
HCOOH (122.4)	NaOH (49.6)	3.5	3.483	72.7	6.5
HCOOH (72.9)	NaOH (49.9)	4.0	4.009	37.3	6.5
CH_3COOH (123.0)	NaOH (50)	4.5	4.500	72.3	8.0
CH_3COOH (73.1)	NaOH (50)	5.0	4.992	37.3	8.0
CH_3COOH (57.3)	NaOH (50)	5.5	5.461	14.7	8.0
MES (104.7)	NaOH (50)	6.0	5.991	63.0	8.0
MES (67.3)	NaOH (50)	6.5	6.423	30.1	8.0
MOPS (114.0)	NaOH (50)	7.0	6.978	39.3	8.0
MOPS (70.2)	NaOH (50)	7.5	7.470	15.8	8.0
MOPS (56.4)	NaOH (50)	8.0	7.934	5.4	8.0
tricine (68.1)	NaOH (50)	8.5	8.472	31.1	8.0
tricine (55.7)	NaOH (50)	9.0	8.928	11.8	8.0
CHES (75.4)	NaOH (50)	9.5	9.613	53.6	8.0
CHES (57.9)	NaOH (49.9)	10.0	10.077	24.0	8.0
CHES (52.1)	NaOH (49.6)	10.5	10.480	9.5	8.0
H_3PO_4 (14.7)	NaOH (32.2)	11.0	11.072	6.4	8.0
H_3PO_4 (12.0)	NaOH (31.3)	11.5	11.527	15.5	8.0
H_3PO_4 (8.1)	NaOH (33)	12.0	11.962	35.4	6.0

Table S-3. Preparation of buffers at 50 mM ionic strength and applied voltages for the IS-CE method. The preparation of the buffers can be found in the article by Henchoz *et al.*[3]

Buffer composition		Theoretical pH	Measured pH	Buffer capacity	Applied voltage
Buffered species (mM)	Buffering species (mM)	(25°C)	(25°C)	(mM / pH unit)	(kV)
H_3PO_4 (41.2)	NaOH (45.6)	6.0	5.987	9.5	8.0
H_3PO_4 (103.9)	NaOH (37.7)	2.0	1.988	89.3	5.0

S-7 SUPPLEMENTARY INFORMATION – FIGURES S-1 – S-10

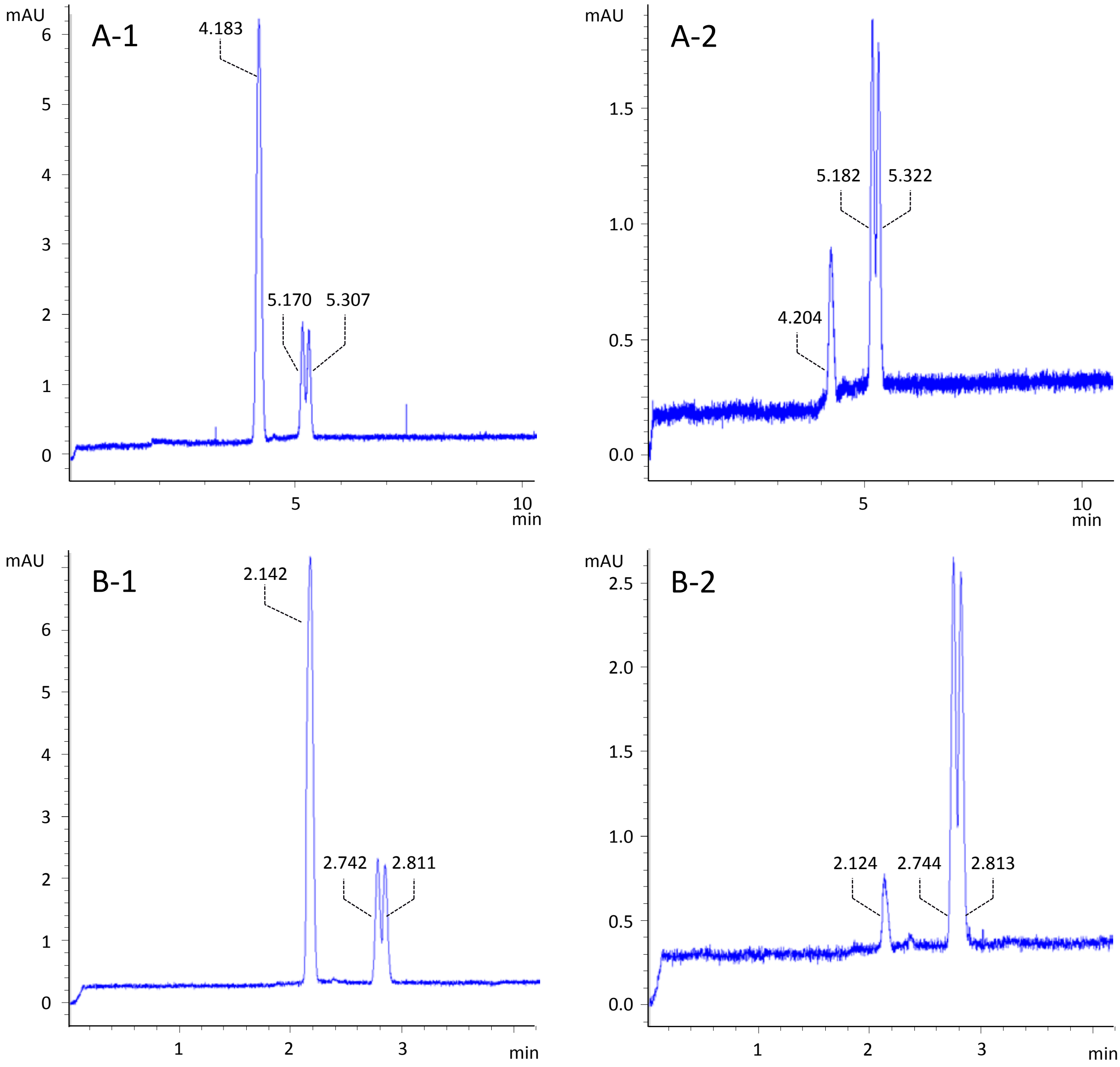

Figure S-1. Electropherograms obtained with the IS-CE approach. (A-1) α2,3-sialyllactose, α2,6-sialyllactose, and neutral marker at pH 6.0; **(A-2)** α2,3-sialyllactose and α2,6-sialyllactose (no neutral marker) at pH 6.0; **(B-1)** α2,3-sialyllactose, α2,6-sialyllactose, and neutral marker at pH 2.0; **(B-2)** α2,3-sialyllactose and α2,6-sialyllactose (no neutral marker) at pH 2.0.

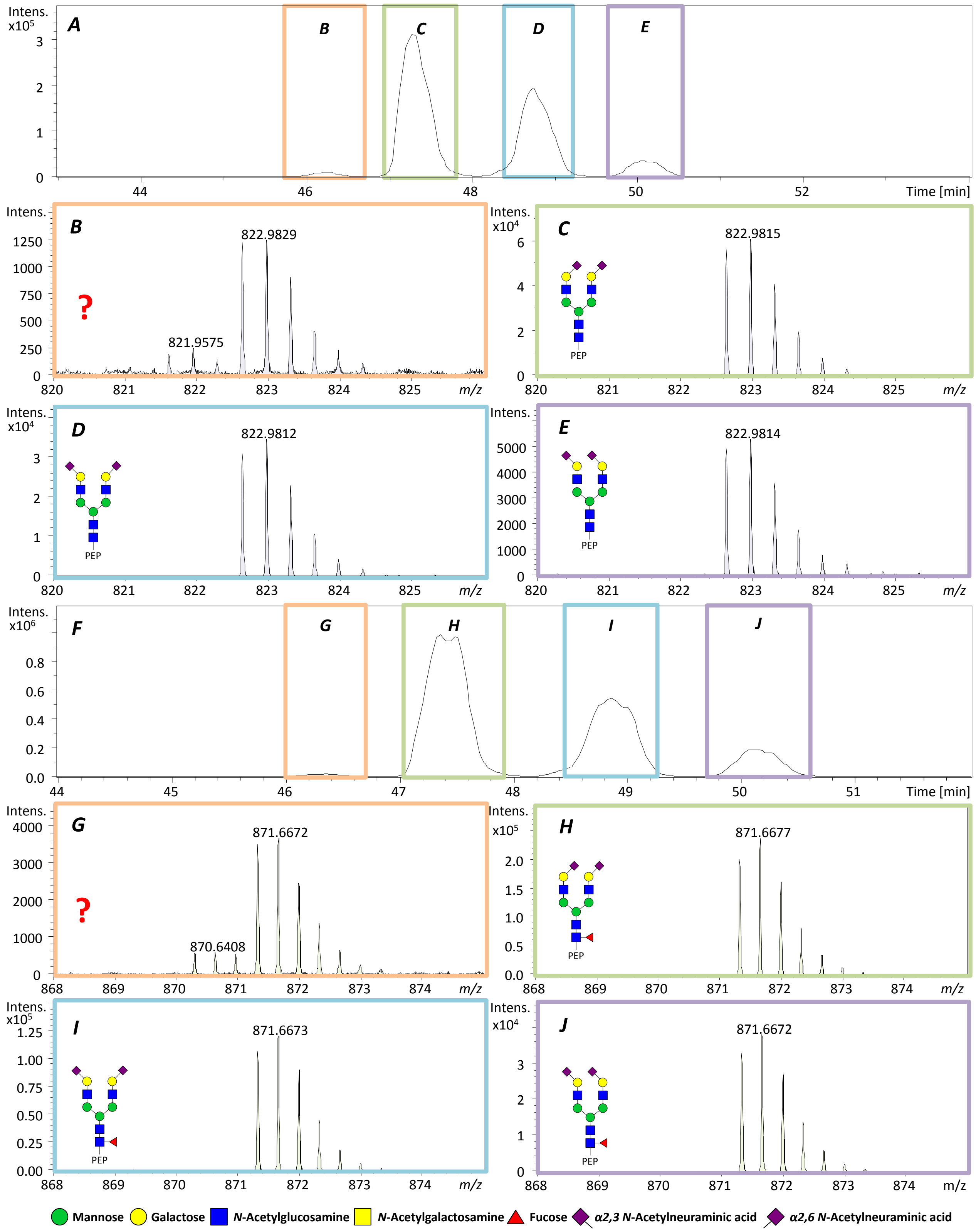

Figure S-2. Isotopic pattern of di-sialylated tryptic glycopeptides at glycosylation sites N_{69} from PSA analysed with CE–ESI-MS. The extracted ion electropherograms of H5N4S2 **(A)** and H5N4F1S2 **(F)**. Isotopic pattern of an unassigned glycopeptide **(B)**, H5N4$S_{2,6}$2 **(C)**, H5N4$S_{2,6}S_{2,3}$ **(D)**, H5N4$S_{2,3}$2 **(E)**, an unassigned glycopeptide **(G)**, H5N4F1$S_{2,6}$2 **(H)**, H4N5F1$S_{2,6}S_{2,3}$ **(I)** and H5N4F1$S_{2,3}$2 **(J)**.

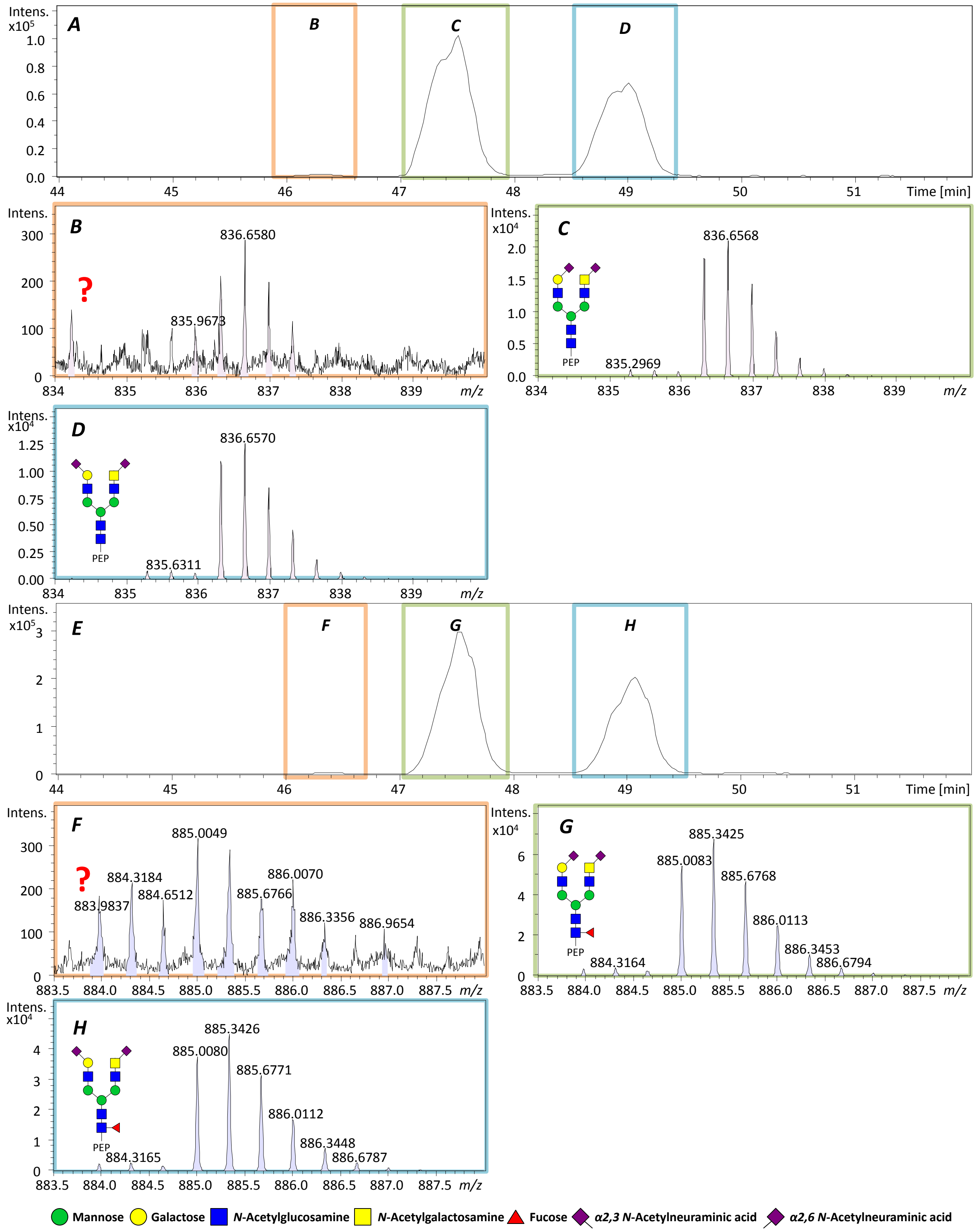

Figure S-3. Isotopic pattern of di-sialylated tryptic glycopeptides at glycosylation sites N_{69} from PSA analysed with CE–ESI-MS. The extracted ion electropherograms of H4N5S2 **(A)** and H4N5F1S2 **(E)**. Isotopic pattern of an unassigned glycopeptide **(B)**, H4N5$S_{2,6}$2 **(C)**, H4N5$S_{2,6}S_{2,3}$ **(D)**, an unassigned glycopeptide **(F)**, H5N4F1$S_{2,6}$2 **(G)** and H4N5F1$S_{2,6}S_{2,3}$ **(H)** .

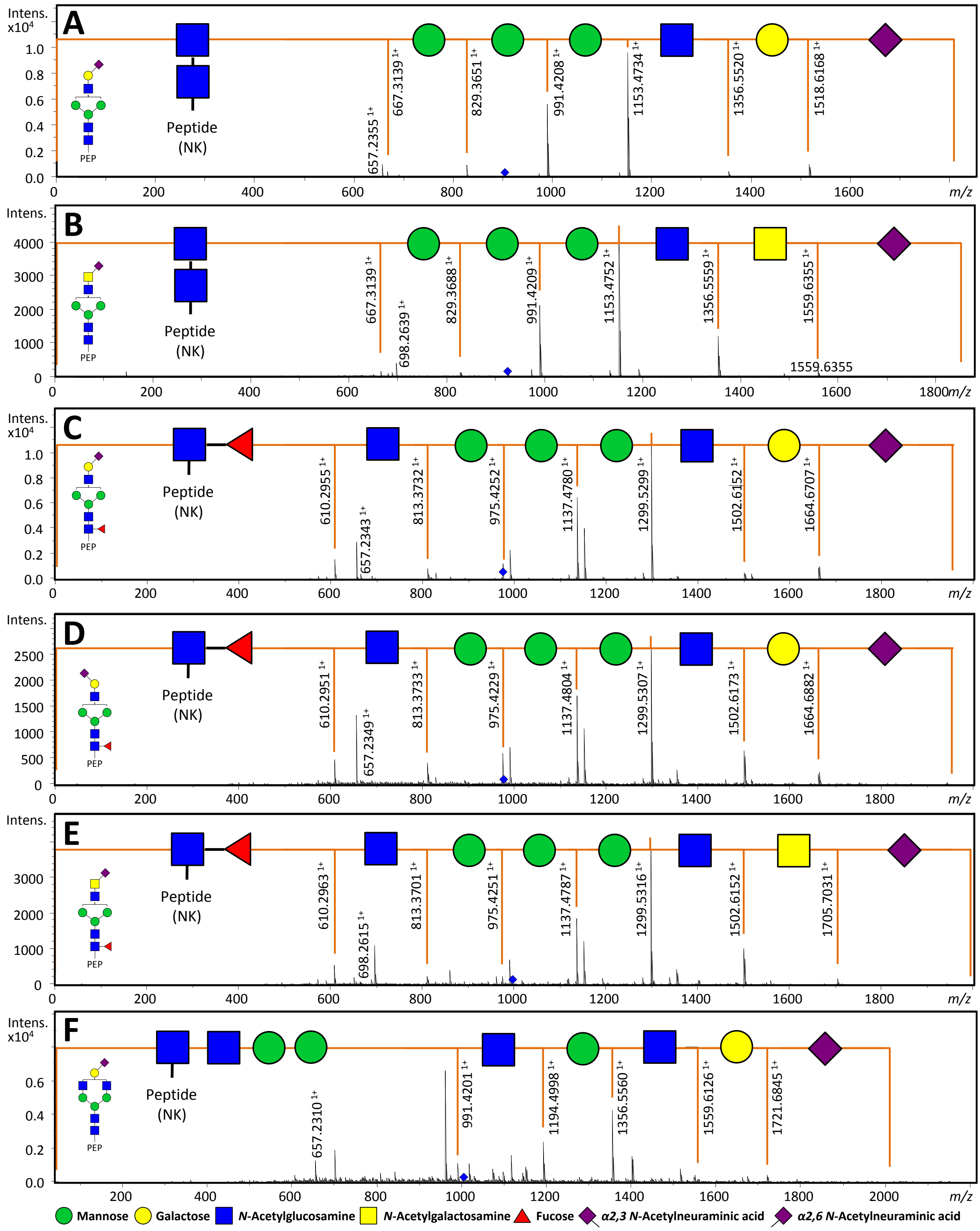

Figure S-4. Fragmentation spectra of mono-sialylated tryptic glycopeptides at glycosylation sites N_{69} from PSA analysed with CE–ESI-MS. The following precursor ions were fragmented **(A)** 905.3536 $^{2+}$ (H4N3$S_{2,6}$1), **(B)** 925.8672 $^{2+}$ (H3N4$S_{2,6}$1) **(C)** 977.4336 $^{2+}$ (H4N3F1$S_{2,6}$1), **(D)** 978.3825 $^{2+}$ (H4N3F1$S_{2,3}$1), **(E)** 999.0279 $^{2+}$ (H3N4F1$S_{2,6}$1) and **(F)** 1006.8925 $^{2+}$ (H4N4F1$S_{2,6}$1). Blue diamond illustrates the precursor ion, PEP illustrates the tryptic peptide sequence **N**K.

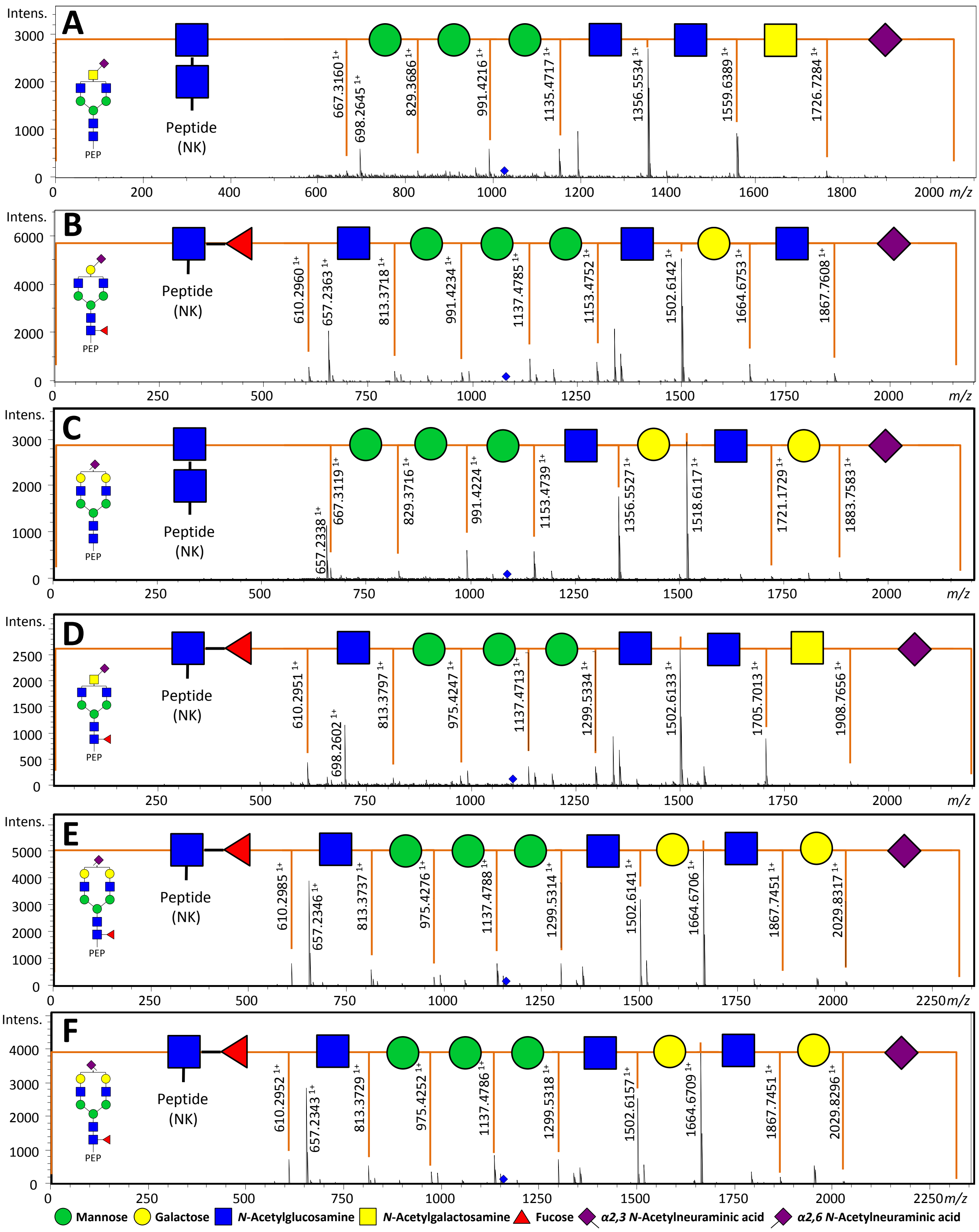

Figure S-5. Fragmentation spectra of mono-sialylated tryptic glycopeptides at glycosylation sites N_{69} from PSA analysed with CE–ESI-MS. The following precursor ions were fragmented **(A)** 1027.4062 $^{2+}$ (H3N5S$_{2,6}$1), **(B)** 1080.2912 $^{2+}$ (H4N4F1S$_{2,6}$1) **(C)** 1088.1100 $^{2+}$ (H5N4S$_{2,6}$1), **(D)** 1100.4350 $^{2+}$ (H4N3F1S$_{2,3}$1), **(E)** 1161.2845 $^{2+}$ (H3N4F1S$_{2,6}$1) and **(F)** 1161.2845 $^{2+}$ (H4N4F1S$_{2,6}$1). Blue diamond illustrates the precursor ion, PEP illustrates the tryptic peptide sequence **N**K.

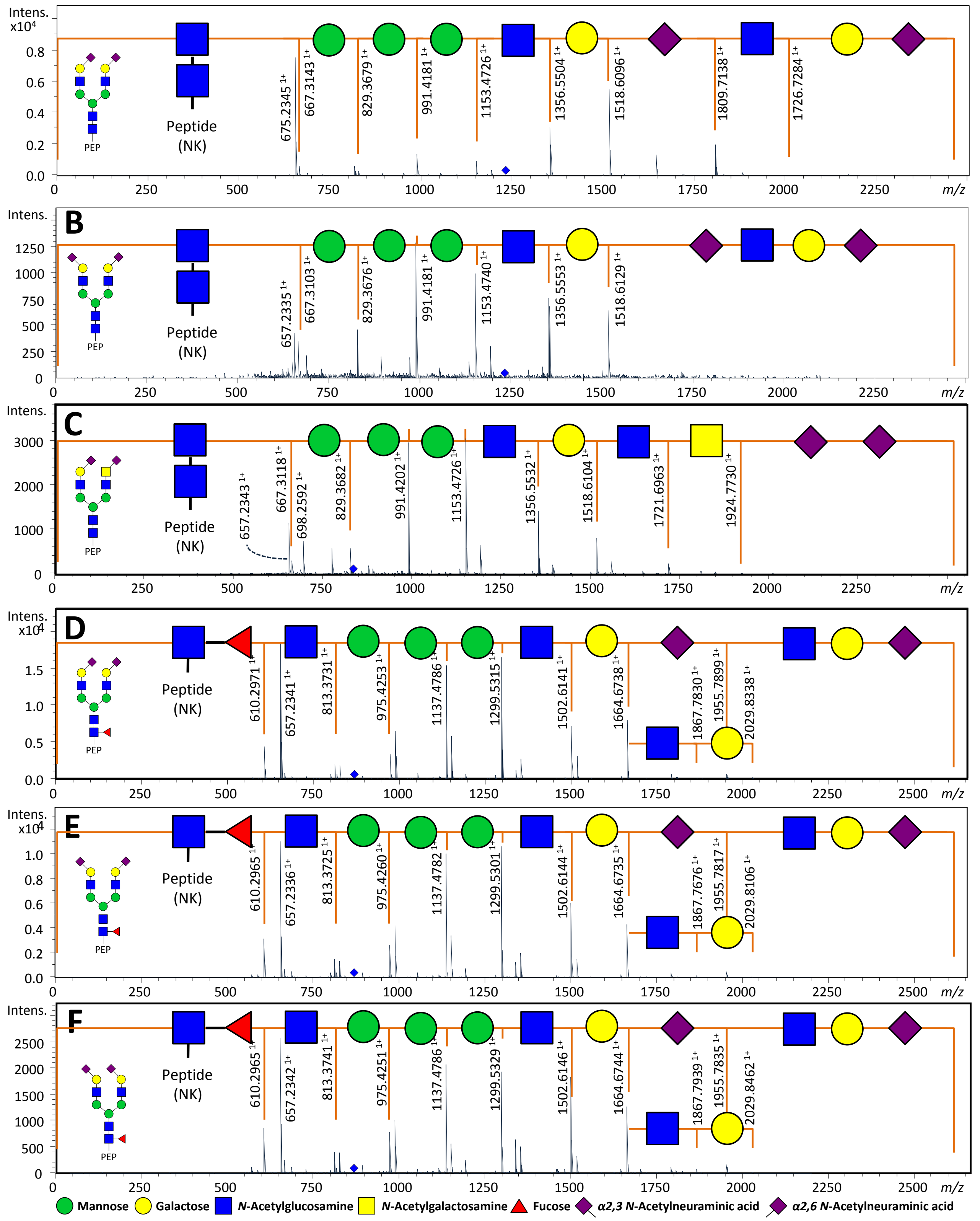

Figure S-6. Fragmentation spectra of di-sialylated tryptic glycopeptides at glycosylation sites N_{69} from PSA analysed with CE–ESI-MS. The following precursor ions were fragmented **(A)** 1233.9676 $^{2+}$ (H5N4S$_{2,6}$2), **(B)** 1233.9677 $^{2+}$ (H5N4S$_{2,3}$1S$_{2,6}$1) **(C)** 836.6300 $^{3+}$ (H4N5S$_{2,6}$2), **(D)** 871.4509 $^{3+}$ (H5N4F1S$_{2,6}$2), **(E)** 871.6134 $^{3+}$ (H5N4F1S$_{2,3}$1S$_{2,6}$1) and **(F)** 871.6515 $^{3+}$ (H5N4F1S$_{2,3}$2). Blue diamond illustrates the precursor ion, PEP illustrates the tryptic peptide sequence **N**K.

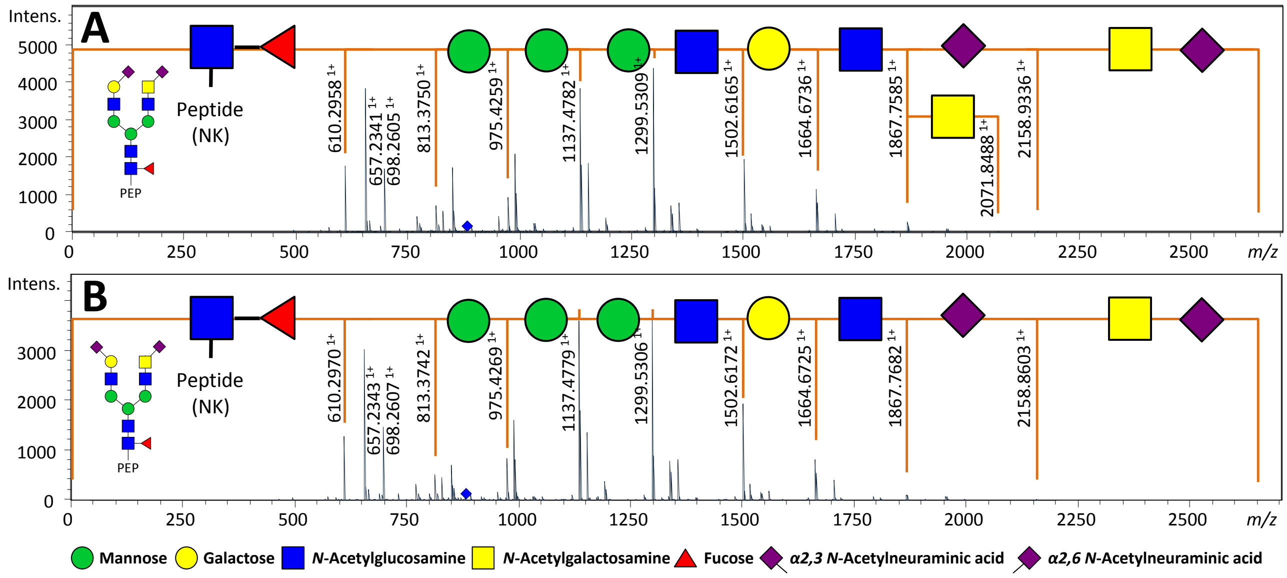

Figure S-7. Fragmentation spectra of di-sialylated tryptic glycopeptides at glycosylation sites N_{69} from PSA analysed with CE–ESI-MS. The following precursor ions were fragmented **(A)** 885.3696 $^{3+}$ (H4N5F1$S_{2,6}$2) and **(B)** 885.3104 $^{3+}$ (H4N5F1$S_{2,3}$1$S_{2,6}$1).Blue diamond illustrates the precursor ion, PEP illustrates the tryptic peptide sequence **N**K.

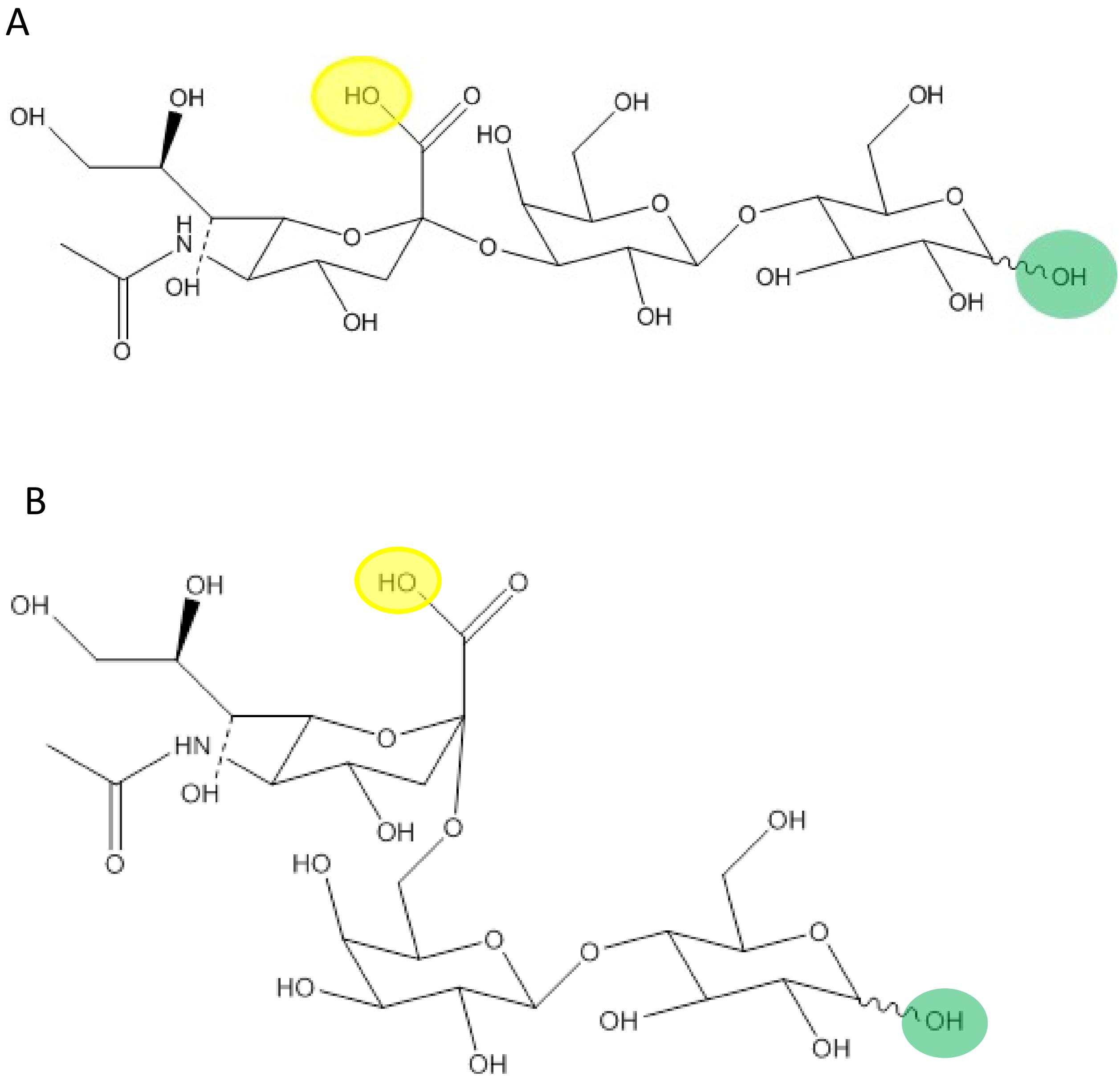

Figure S- 8. Chemical structure of **(A)** α2,3 sialyllactose and **(B)** α2,6 sialyllactose

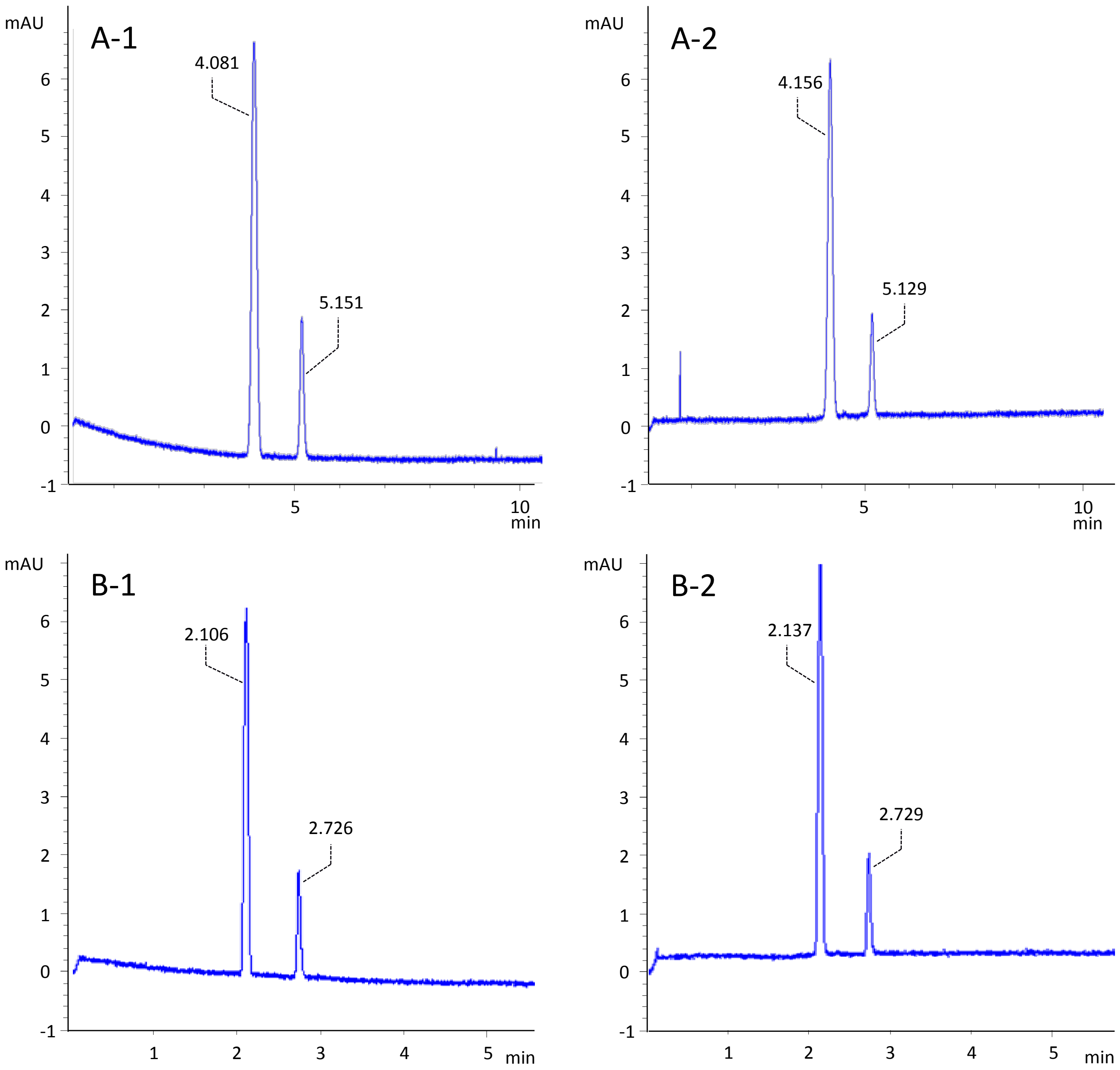

Figure S-9. Electropherograms obtained with the conventional CE approach. (A-1) α2,3-sialyllactose and **(A-2)** α2,6-sialyllactose with a neutral marker at pH 6.0; **(B-1)** α2,3-sialyllactose and **(B-2)** α2,6-sialyllactose with a neutral marker at pH 2.0.

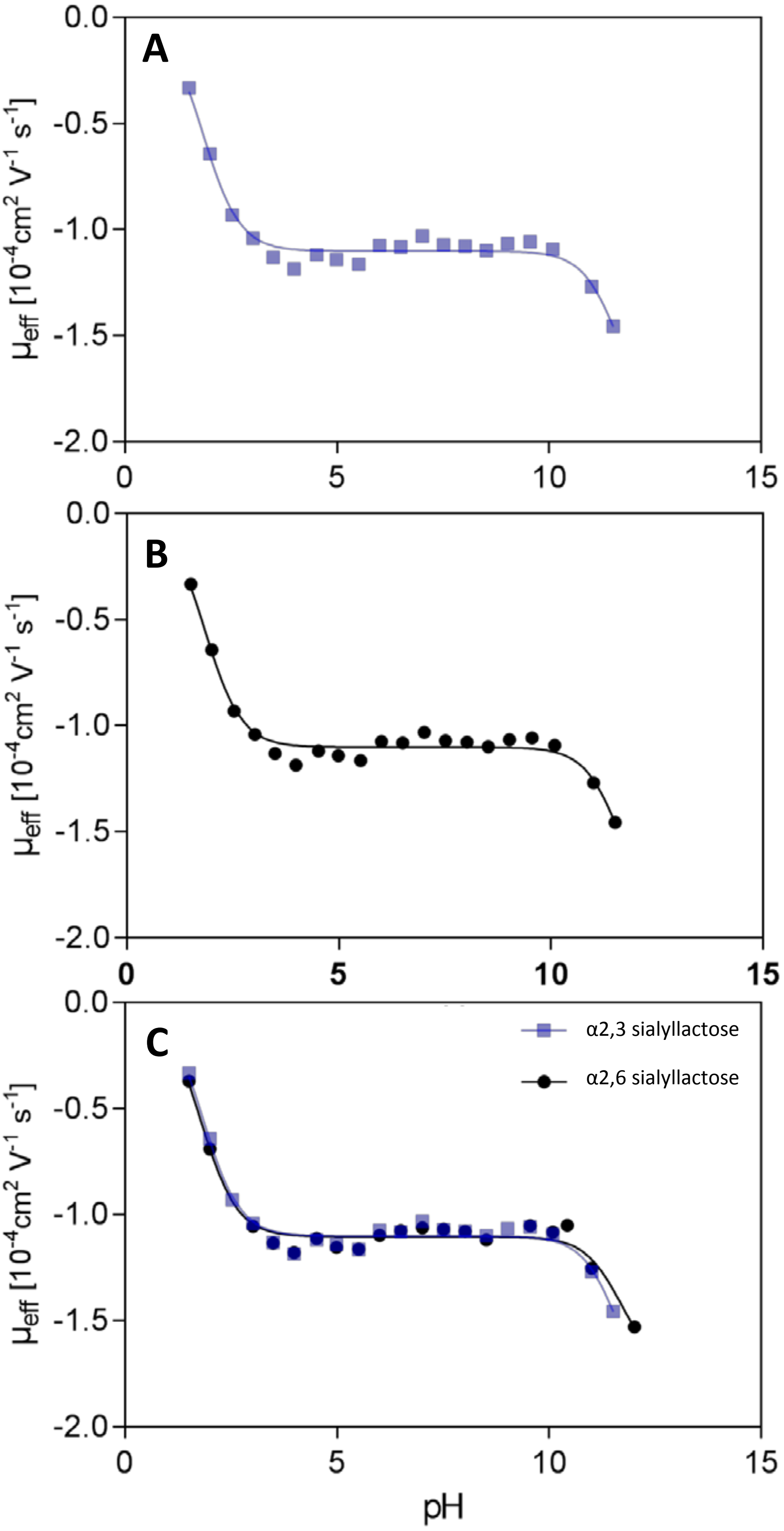

Figure S-10. Relationship between μ_{eff} and pH for **(A)** α2,3 sialyllactose, **(B)** α2,6 sialyllactose and **(C)** an overlay of these two compounds**.**

S-8 REFERENCES

(1) Geiser, L.; Henchoz, Y.; Galland, A.; Carrupt, P.-A.; Veuthey, J.-L. *J. Sep. Sci.* **2005**, *28*, 2374-2380.
(2) Fuguet, E.; Ràfols, C.; Bosch, E.; Rosés, M. *Chem. Biodiversity* **2009**, *6*, 1822-1827.
(3) Henchoz, Y.; Schappler, J.; Geiser, L.; Prat, J.; Carrupt, P.-A.; Veuthey, J.-L. *Anal. Bioanal. Chem.* **2007**, *389*, 1869-1878.
